# Solvent-Dependent Metabolomic Profiles and Antioxidant Properties of the Invasive Seaweed *Caulerpa cylindracea* from the Adriatic Sea

**DOI:** 10.3390/ph19071065

**Published:** 2026-07-10

**Authors:** Ines Kovačić, Iris Peričić, Mariana Jurica, Neven Iveša, Smiljana Goreta Ban, Nikola Major, Josipa Bilić, Gioconda Millotti

**Affiliations:** 1 Faculty for Natural Sciences, Juraj Dobrila University of Pula, 52100 Pula, Croatia; ines.kovacic@unipu.hr (I.K.); ipericic@unipu.hr (I.P.); mjurica@student.unipu.hr (M.J.); neven.ivesa@unipu.hr (N.I.); 2 Department of Agriculture and Nutrition, Institute of Agriculture and Tourism, Carlo Hugues 8, 52440 Porec, Croatia; smilja@iptpo.hr (S.G.B.); nikola@iptpo.hr (N.M.); 3 The Centre of Excellence for Biodiversity and Molecular Plant Breeding, Svetošimunska 25, 10000 Zagreb, Croatia; 4 METRIS Research Centre, Istrian University of Applied Sciences, Preradovićeva 9D, 52100 Pula, Croatia; jbilic@iv.hr

**Keywords:** green algae, non-indigenous species, bioactive metabolites, phytochemical profiling, radical-scavenging activity

## Abstract

**Background/Objectives**: Widely distributed in the Adriatic Sea, the invasive green alga *Caulerpa cylindracea* poses ecological risks but also constitutes a largely untapped source of bioactive compounds. This study aimed to characterize its metabolomic profile, phenolic composition, and antioxidant capacity to explore its potential for biotechnological use. **Methods**: Samples from the Northern Adriatic Sea were extracted with water, 70% ethanol, and 80% methanol. Phenolic compounds were analyzed by LC-QqQ, while untargeted metabolomic profiling was performed using LC-qTOF. Total phenolic, flavonoid, and non-flavonoid contents were determined spectrophotometrically, and antioxidant activity was evaluated using DPPH, ABTS, and FRAP assays. **Results**: Metabolomic profiling demonstrated a clear solvent-dependent differentiation, with aqueous extracts enriched in polar metabolites, while organic extracts contained higher levels of lipid-derived and secondary metabolites. Antioxidant assays indicated that aqueous extracts exhibited the strongest radical-scavenging activity (DPPH and ABTS), whereas the highest reducing capacity (FRAP) was observed in ethanolic extracts. In addition, total flavonoid content was greatest in the ethanol extracts. **Conclusions**: These results highlight its potential for valorization as a sustainable resource in food, cosmetic, and biomedical applications, while also supporting approaches for managing its spread.

## 1. Introduction

Scientific research groups have recently shown growing interest in organisms living in aquatic habitats, studying their pharmaceutical and biomedical properties. These studies have highlighted the diverse bioactive compounds in these organisms, including their antioxidant, anti-inflammatory, antifungal, antibacterial, and neuroprotective activities [1]. Seaweeds are often subjected to harsh environmental conditions, and the resulting damage is not always immediately visible. As a result, seaweeds produce a variety of metabolites to protect themselves from abiotic stressors such as herbivory and mechanical damage from the sea [1].

Caulerpaceae is a family of green algae represented by a single, distinctive genus, *Caulerpa*. This genus includes 101 species, along with 40 varieties and 67 forms recorded in southern Australia [2]. They typically grow in shallow, tropical and subtropical marine habitats *Caulerpa cylindracea* Sonder is a species of seaweed native to the Australian coast, found across a wide range from Perth northward along Western Australia. It was first introduced to the Mediterranean Sea in the early 1990s, where it has since spread extensively and is now considered an invasive species. While this species has been studied in terms of its taxonomy, invasiveness, and ecological characteristics, there is still a lack of research on its chemical composition, and potential applications for human health.

In the Adriatic Sea, *C. cylindracea* grows rapidly, colonizes diverse habitats, and spreads through fragmentation and propagation, leading to significant structural and functional changes in marine communities and affecting fish metabolism [3] and *Cymodocea nodosa* meadows population [4]. Invasive algae threaten biodiversity, alter ecosystem functions, and impact fisheries and tourism, yet their fast growth and high biomass make them valuable sources of bioactive compounds [5]. Given the impracticality of completely eradicating non-indigenous species, adaptive management and initiatives such as collection and recycling initiatives [6,7,8] are essential to limit their further spread while exploiting these species for applications in nutrition, pharmaceuticals, and cosmeceuticals. This integrated approach transforms an ecological problem into a potential economic opportunity. In this study, *C. cylindracea* is investigated for its potential applications in the cosmeceutical and biomedical fields as a strategy to manage and reduce its populations.

Free radicals are highly reactive molecules that contribute to the aging process and the pathogenesis of numerous human diseases. Antioxidants are compounds capable of scavenging these radicals, thereby playing a crucial role in mitigating oxidative stress and preventing free radical–induced disorders [9]. Several studies have demonstrated the in vitro antioxidant capacity of seaweeds, which is attributed to the presence of novel compounds such as carotenoids, specific polysaccharides, and polyphenols. These compounds exhibit radical-scavenging activity by neutralizing reactive oxygen species through their own oxidation [5]. This plays a crucial role in protecting against various health conditions, including chronic inflammation, atherosclerosis, cancer, cardiovascular disease, and the aging process [10].

Recently, the nutritional profile of *C. cylindracea* was explored [11,12,13], showing promising potential. Only limited studies have investigated the antioxidant properties of *C. cylindracea* [3,11,13], and comparisons are difficult due to differences in geographic origin and extraction methods or solvents. Its metabolite composition remains largely unknown, and its potential benefits for human health are unexplored.

In this study, the green alga *C. cylindracea*, collected from the North Adriatic Sea, was analyzed for its untargeted metabolomic profile and phenolic composition composition. To the best of our knowledge, no metabolomic profile was studied on this seaweed, and phenolic data are very scarse. The antioxidative capacity of *C. cylindrace* water, ethanol and methanol was also studied. This work aims to advance knowledge of this marine resource and promote its sustainable use. Although specific data on its applications in health and cosmetics are currently limited, its biochemical composition provides a strong basis for potential development in these fields.

## 2. Results

### 2.1. Phenolic Content and Antioxidative Capacity

Overall, the results indicate a clear effect of solvents among the extracts of *C. cylindracea* (Table 1).

#### 2.1.1. Total Phenolic Content

**Figure 1:**
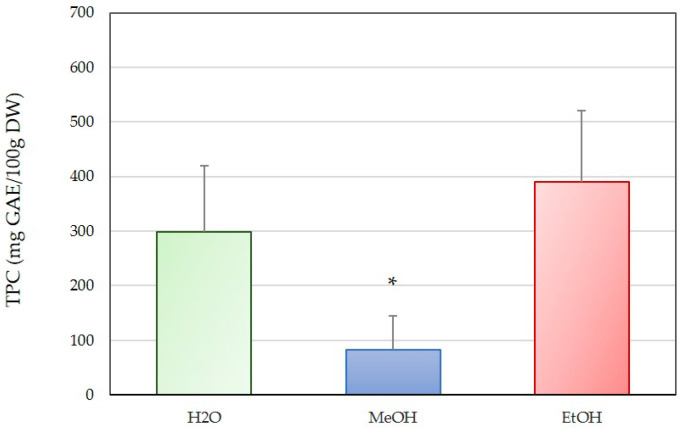
Total phenolic content (TPC) of aqueous (H_2_O), methanol (MeOH) and ethanol (EtOH) extract of *C. cylindracea* from the Northern Adriatic. Values are expressed as average ± standard deviation. Stars (*) indicate a significant difference at the level of *p* ≤ 0.05 calculated with the Mann-Whitney test.

The total phenolic content (TPC, Figure 1) of *C. cylindracea* varied markedly depending on the extraction solvent (Kruskal Wallis test, Table 1). The ethanol (EtOH) extract exhibited the highest TPC, with an average value of 389.78 mg GAE/100 g DW, indicating that ethanol was the most effective solvent for extracting phenolic compounds from this species. The aqueous (H_2_O) extract showed a moderately high TPC of 298.67 GAE/100 g DW. In contrast, the methanol (MeOH) extract yielded the lowest TPC, with an average of 82.96 mg GAE/100 g DW, and was statistically different from the water and ethanol extracts (Mann-Whitney, *p* < 0.05).

#### 2.1.2. Total Flavonoid Content

**Figure 2:**
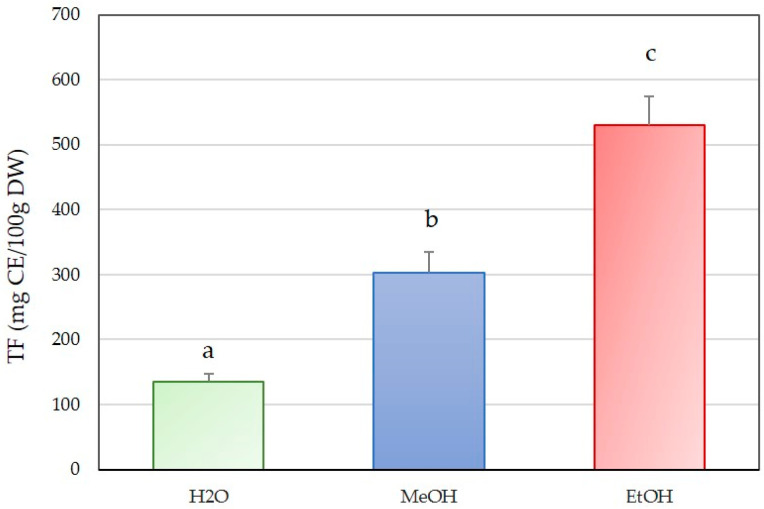
Total flavonoids (TF) of aqueous (H_2_O), methanol (MeOH) and ethanol (EtOH) extract of *C. cylindraceae* from the Northern Adriatic. Values are expressed as average ± standard deviation. Different letters indicate a significant difference at the level of *p* ≤ 0.05 calculated with the Mann-Whitney test.

The total flavonoid content (TF, Figure 2) of *C. cylindracea* extracts differed substantially among the solvents tested (Kruskal-Wallis test, Table 1). The ethanol (EtOH) extract showed the highest TF, with an average value of 530.75 CE/100 g DW, and significantly different to other two solvents (Mann-Whitney test, *p* ≤ 0.05). The methanol (MeOH) extract exhibited an intermediate TF of 302.17 mg CE/100 g DW, and sig-nificantly different to other two solvents (Mann-Whitney test, *p* ≤ 0.05). In contrast, the aqueous (H_2_O) extract presented the lowest TF, with an average of 135.59 mg CE/100 g DW, and significantly different to other two solvents (Mann-Whitney test, *p* ≤ 0.05).

#### 2.1.3. Total Non-Flavonoids

**Figure 3:**
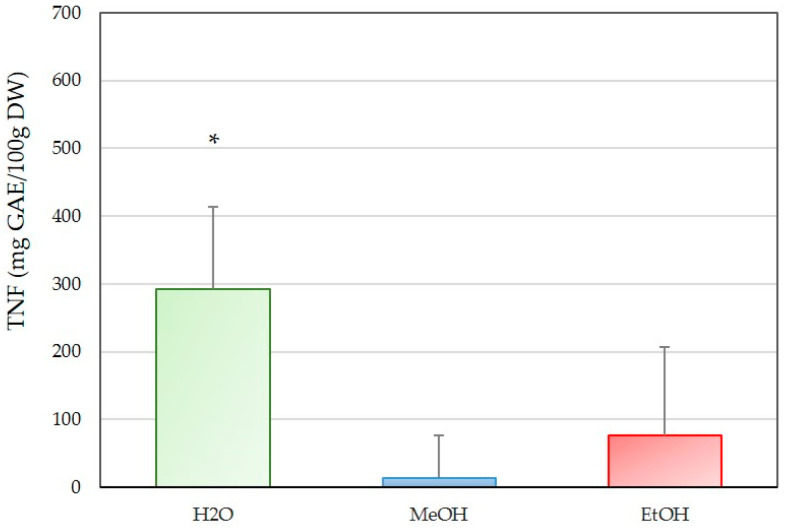
Total non-flavonoids (TNF) of aqueous (H_2_O), methanol (MeOH) and ethanol (EtOH) extract of *C. cylindracea* from the Northern Adriatic. Values are expressed as average ± standard deviation. Stars (*) indicate a significant difference at the level of *p* ≤ 0.05 calculated with the Mann-Whitney test.

The total non-flavonoids (TNF, Figure 3) of *C. cylindracea* extracts showed clear differences depending on the solvent used (Kruskal-Wallis test, Table 1. The aqueous (H_2_O) extract exhibited the highest TNF, with an average value of 292.73 mg GAE/100 g DW, indicating that water was the most effective solvent compared to methanol and ethanol extracts (Mann-Whitney test, *p* < 0.05). The ethanol (EtOH) extract showed a moderate TNF value of 76.62 mg GAE/100 g DW, while the methanol (MeOH) extract yielded the lowest TNF, with an average of 13.62 mg GAE/100 g DW.

#### 2.1.4. Antioxidative Properties

The results indicate a clear effect of solvents on the antioxidative content measured in extracts of *C. cylindracea* with ABTS, FRAP and DPPH test (Table 2).

**Figure 4:**
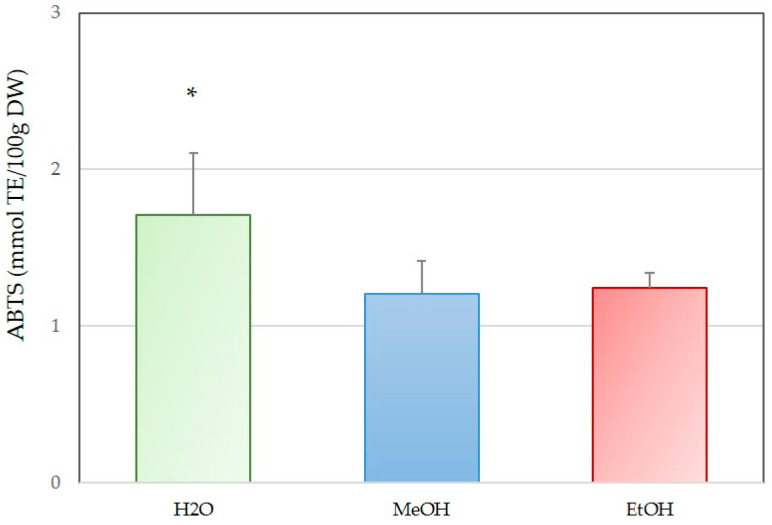
Antioxidant capacity of aqueous (H_2_O), methanol (MeOH) and ethanol (EtOH) extract of *C. cylindracea* from the Northern Adriatic measured by ABTS. Values are expressed as average ± standard deviation. Stars (*) indicate a significant difference at the level of *p* ≤ 0.05 calculated with the Tukey HSD test.

The antioxidant capacity of extracts, as determined by the ABTS assay (Figure 4), showed significant variation among the solvents (one-way ANOVA test, Table 2). The aqueous (H_2_O) extract exhibited the highest ABTS radical scavenging activity and was significantly different to water and methanol extracts (Tukey HSD test, *p* < 0.05), with an average value of 1.702 mmol TE/100 g DW. The ethanol (EtOH) extract showed a slightly lower activity of 1.239 mmol TE/100 g DW, while the methanol (MeOH) extract presented the lowest value at 1.201 mmol TE/100 g DW.

**Figure 5:**
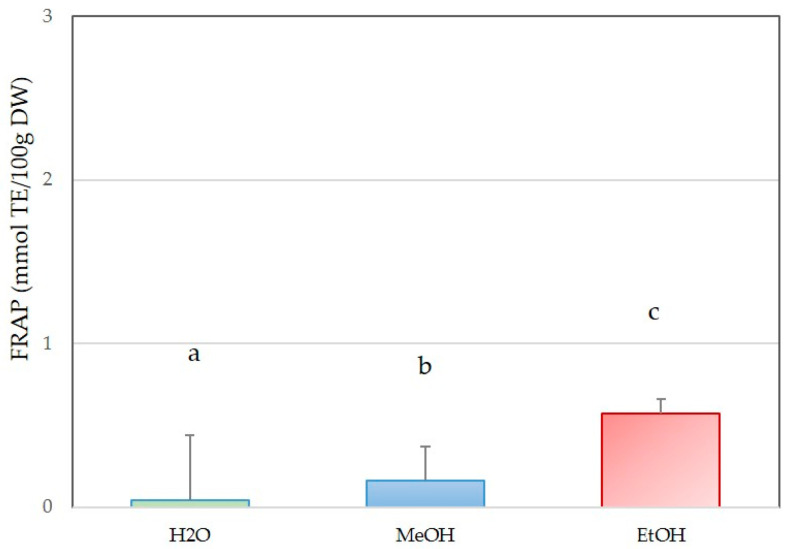
Antioxidant capacity of aqueous (H_2_O), methanol (MeOH) and ethanol (EtOH) extract of *C. cylindracea* from the Northern Adriatic measured by FRAP. Values are expressed as average ± standard deviation. Different letters indicate a significant difference at the level of *p* ≤ 0.05 calculated with the Tukey HSD test.

The antioxidant capacity of *C. cylindracea* extracts, as measured by the FRAP assay (Figure 5), differed markedly among the solvents (one-way ANOVA test, Table 2). The ethanol (EtOH) extract exhibited the highest ferric reducing antioxidant power compared to other extracts (Tuckey HSD test, *p* < 0.05), with an average value of 0.567 mmol TE/100 g DW. The methanol (MeOH) extract showed a moderate FRAP value of 0.161 mmol TE/100 g DW, while the aqueous (H_2_O) extract displayed the significantly lowest FRAP value of 0.039 mmol TE/100 g DW (Tuckey HSD test, *p* < 0.05).

**Figure 6:**
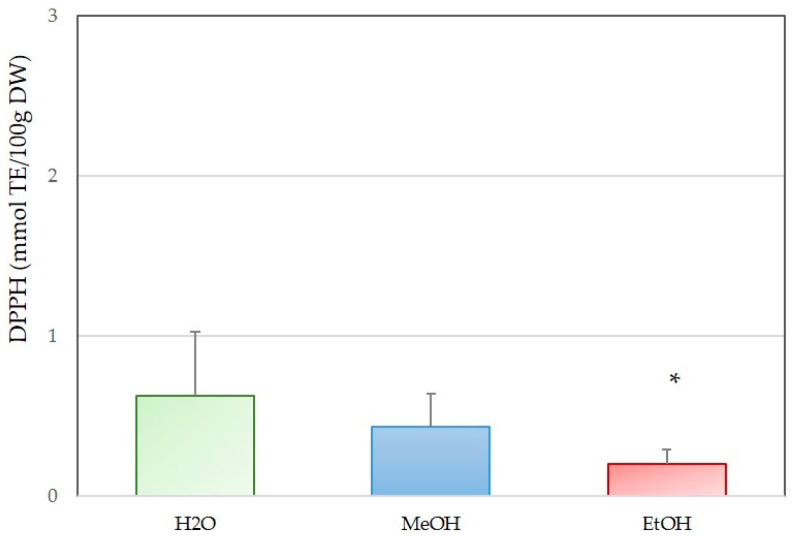
Antioxidant capacity of aqueous (H_2_O), methanol (MeOH) and ethanol (EtOH) extract of *C. cylindracea* from the Northern Adriatic measured by DPPH. Values are expressed as average ± standard deviation. Stars (*) indicate significant difference at the level of *p* ≤ 0.05 calculated with Tukey HSD test.

The antioxidant capacity of *C. cylindracea* extracts, as evaluated by the DPPH radical scavenging assay (Figure 6), showed noticeable differences among the solvents (one-way ANOVA test, Table 2). The aqueous (H_2_O) extract exhibited the highest DPPH scavenging activity, with an average value of 0.624 mmol TE/100 g DW. The methanol (MeOH) extract showed a moderate activity of 0.429 mmol TE/100 g DW, while the ethanol (EtOH) extract presented the lowest value at 0.197 mmol TE/100 g DW, and significantly different to other two extracts (Tuckey HSD test, *p* < 0.05).

**Figure 7:**
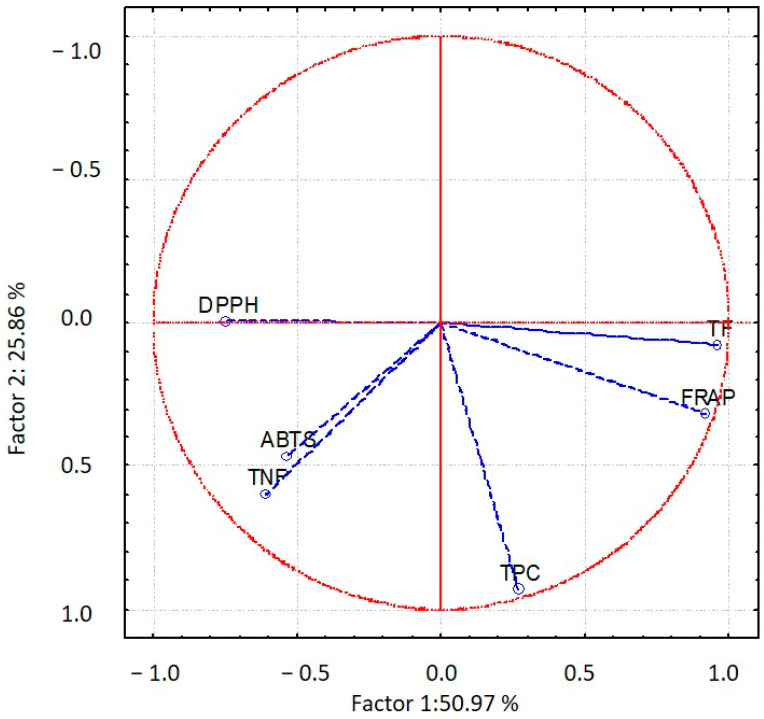
Principal component analysis (PCA) of antioxidant activity of *C. cylindracea* from the Northern Adriatic measured by ABTS, FRAP and DPPH assays and total phenolic content (TPC), total flavonoids (TF) and total non-flavonoids (TNF).

For *C. cylindraceae*, the first two components in Figure 7 explained 76.83% of the total variance (PC1: 50.97%, PC2: 25.86%). The correlation structure used in PCA shows that FRAP is strongly positively related to TF (0.956), modestly to TPC (0.518), forming a common component of reducing power. This component is negatively associated with DPPH, indicating opposing antioxidant mechanisms. TNF aligns partly with DPPH (0.480) and ABTS (0.326), contributing to a secondary component.

### 2.2. Chemical Composition of Phenolic Compounds of C. cylindracea

The detected phenolic compounds of *C. cylindracea* through targeted LC-QqQ profiling are given in Table 3. In total, 7 compounds were identified in the ethanolic and methanolic extracts. No compound was detected in the water extract. The concentrations of the detected compounds were generally comparable between the ethanolic and methanolic extracts, although two of the seven compounds were significantly more abundant in the methanolic extract (sarsasapogenin and Luteolin-4′-O-glucoside).

**Figure 8:**
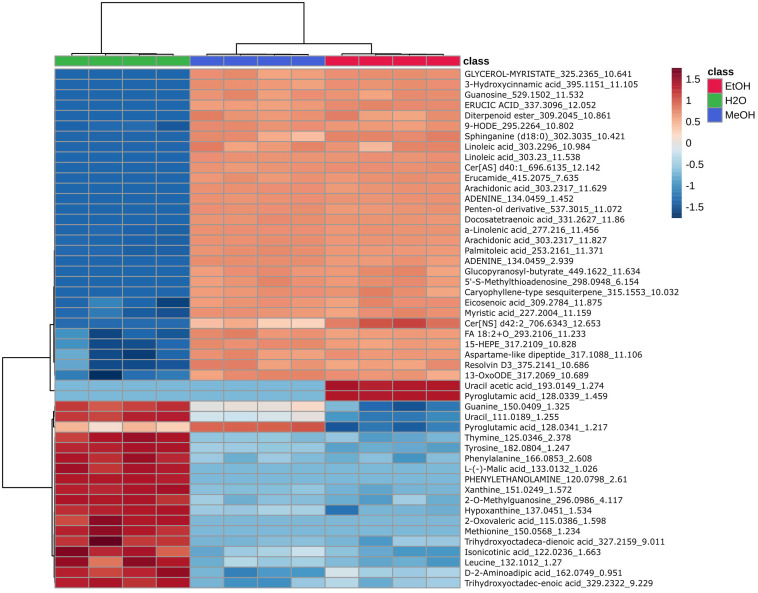
Heatmap representing the relative abundance of the metabolites of aqueous (H_2_O), methanol (MeOH) and ethanol (EtOH) extract of *C. cylindracea* from the Northern Adriatic which showed significant differences with ANOVA in relation to the extraction solvent used (water, ethanol 70% and methanol 80%). Red indicated higher abundance while blue indicates lower abundance.

Compounds found via an untargeted metabolomic approach using LC-qTOF are presented in Supplementary Table S1. The obtained heatmap (Figure 8) revealed a clear separation of samples according to the extraction solvent, indicating a strong influence of solvent polarity on metabolite composition. Aqueous extracts were characterized by higher levels of polar metabolites, such as amino acids and organic acids. Ethanol and methanol extracts were enriched in less polar compounds, including fatty acids and secondary metabolites.

**Figure 9:**
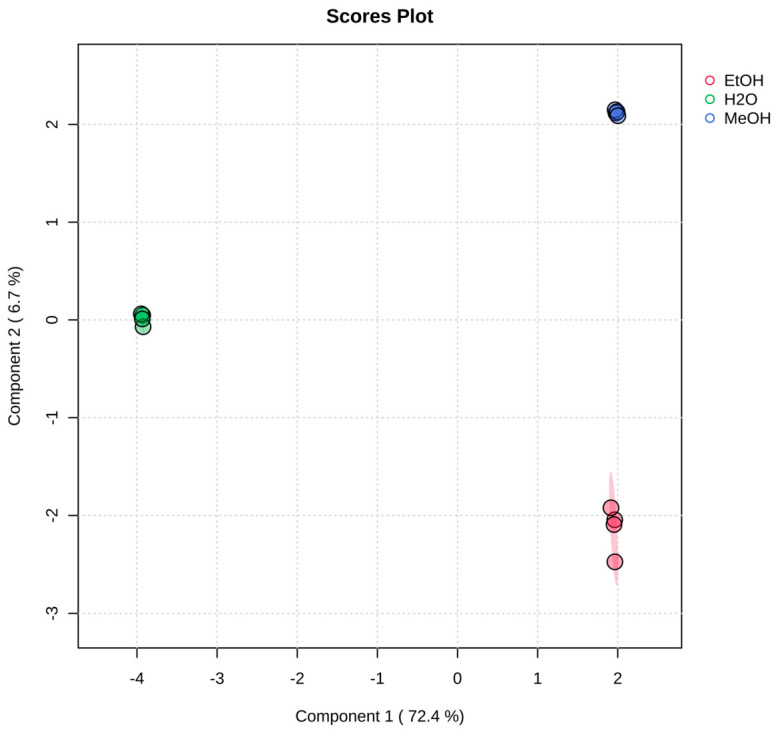
Partial least squares discriminant analysis (PLS-DA) score plot showing the separation of aqueous (H_2_O), methanol (MeOH) and ethanol (EtOH) extract of *C. cylindracea* from the Northern Adriatic based on metabolomic profiles. Component 1 (72.4%) primarily discriminates aqueous extracts, while Component 2 (6.7%) differentiates ethanol and methanol extracts.

Partial least squares discriminant analysis (PLS-DA) (Figure 9) demonstrated a clear segregation of samples based on the extraction solvent, reflecting distinct metabolomic profiles. Component 1, accounting for 72.4% of the variance, primarily separated aqueous extracts from organic extracts, emphasizing the strong effect of solvent polarity. Component 2 (6.7%) further discriminated between ethanol and methanol extracts. The tight clustering of replicates within each group indicates high analytical reproducibility. Overall, these findings confirm that the choice of extraction solvent is a key factor shaping metabolite composition.

**Figure 10:**
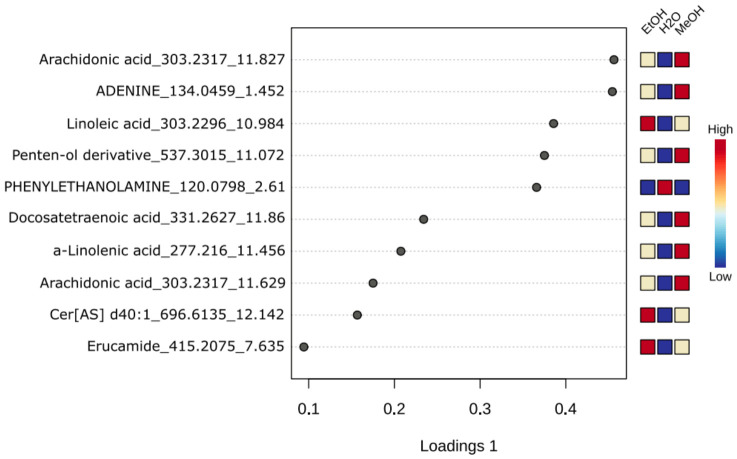
sPLS loading plot for Component 1 showing metabolites of aqueous (H_2_O), methanol (MeOH) and ethanol (EtOH) extract of *C. cylindracea* from the Northern Adriatic contributing to the separation between aqueous and organic extracts.

The sPLS loading plot (Figure 10) highlights the key metabolites contributing to the separation along Component 1. Among the variables with the highest loadings are several fatty acids, lipid-derived compounds, and nucleobases such as adenine. These metabolites are predominantly associated with ethanol and methanol extracts, while their abundance is lower in aqueous extracts. This pattern indicates that organic solvents are more effective in extracting lipid-derived and other bioactive metabolites.

**Figure 11:**
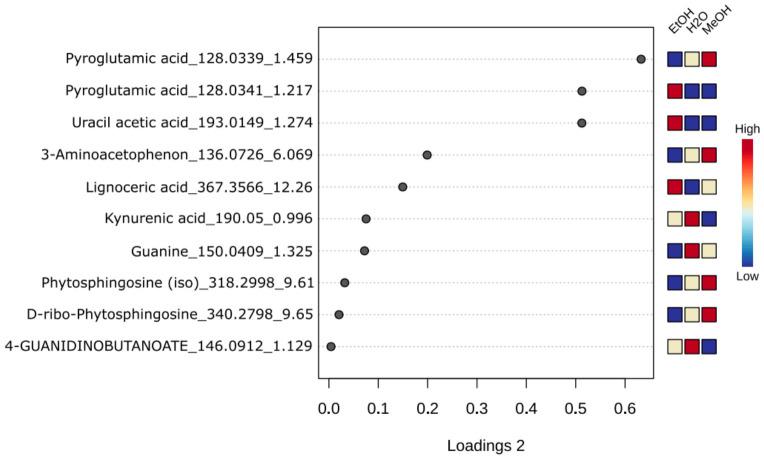
sPLS loading plot for Component 2 showing metabolites of aqueous (H_2_O), methanol (MeOH) and ethanol (EtOH) extract of *C. cylindracea* from the Northern Adriatic contributing to the separation between ethanolic and methanolic extracts.

The sPLS plot for Component 2 (Figure 11) revealed the metabolites driving the differentiation between ethanol and methanol extracts. Key contributors included pyroglutamic acid, nucleobases and their derivatives (e.g., guanine and uracil), nicotinic acid, and lipid-associated compounds such as phosphatidylglycerols. These findings indicate that even closely related solvents, such as ethanol and methanol, exhibit distinct extraction selectivity.

## 3. Discussion

Despite the ecological relevance of *C. cylindracea* as an invasive species in the Mediterranean, information on its metabolomic composition and phenolic profile remains limited. Some secondary metabolites, including caulerpin, monomethyl caulerpinate, β-sitosterol, and palmitic acid were previously reported [14] as well as some volatile bioactive compounds [15]. Solvent polarity strongly influences polyphenol extraction efficiency [16]. When selecting extraction solvents, safety is crucial. Water is the safest solvent but may be less effective in extracting a wide range of bioactive compounds, whereas ethanol, classified as GRAS, offers a favorable balance between extraction efficiency and safety for food and pharmaceutical applications [17]. Methanol is often considered more efficient for phenolic extraction because of its higher polarity [18].

Targeted polyphenolic analysis confirmed the presence of seven phenolic compounds in the ethanol and methanol extracts, whereas none were detected in the water extract. This is consistent with the greater solubility of phenolic compounds in organic solvents such as ethanol and methanol compared with water [19]. Among the identified compounds, 4-hydroxybenzoic acid was the most abundant, followed by 2-hydroxybenzoic acid, vanillin, scoparone, diosgenin, sarsasapogenin, and luteolin-4′-O-glucoside. These compounds have previously been reported in the literature as bioactive plant metabolites with diverse biological properties [20,21,22,23,24,25,26,27,28,29,30]. The occurrence of these metabolites highlights the chemical complexity of *C. cylindracea* extracts and supports their potential as a source of bioactive compounds.

Ouahabi recently reported the phenolic composition of *Caulerpa prolifera*, along the coast of Morocco, identifying 19 compounds in the ethyl acetate and methanol extracts. In the methanolic extract, only two compounds—4-hydroxybenzoic acid and vanillin—were common, while luteolin was detected in *C. cylindracea* in the form of luteolin-4′-O-glucoside [31]. Zhong et al. [32] identified 15 phenolic compounds in *Caulerpa* species from the Australian coast, none of which were found in *C. cylindracea*. This highlights the influence of both species-specific metabolism and geographical factors on the chemical composition of *Caulerpa*, emphasizing the variability of phenolic profiles across locations and species.

Untargeted metabolite analysis using LC-qTOF revealed a clear solvent-dependent extraction pattern and highlighted the chemical diversity of *C. cylindracea* extracts. For example, tentative annotations included resolvin D3, EPA-derived oxygenated lipid mediators, erucic acid, sphinganine-related compounds, linoleic acid, and α-linolenic acid, all of which belong to metabolite classes that have been linked to anti-inflammatory, barrier-supportive, or antioxidant functions in previous studies [33,34,35,36,37,38]. However, as these annotations were assigned at MSI level 2 and were not confirmed using authentic reference standards, no direct conclusions regarding their presence, abundance, or biological activity in *C. cylindracea* extracts can be drawn. Consequently, these observations should be considered preliminary and hypothesis-generating, providing a basis for future targeted analytical and functional studies.

The resulting heatmap demonstrates that the choice of extraction solvent strongly influences the metabolomic profile of *C. cylindracea*. Both hierarchical clustering and PLS-DA analyses show a distinct grouping of samples based on the solvent used, indicating that solvent polarity is the key factor governing metabolite composition. The pronounced separation along component 1 further confirms that aqueous extracts are substantially different from organic extracts. This observation aligns with findings reported in previous studies [39]. The aqueous extract was mainly enriched in polar metabolites, including amino acids and low-molecular-weight organic acids, known to exhibit antioxidant properties through radical scavenging and metal chelating mechanisms [40,41]. The ethanol and methanol extracts contained higher levels of lipid-derived and moderately polar compounds, such as fatty acids, recognized for their antioxidant, antimicrobial, and anti-inflammatory properties [42]. This is consistent with the greater efficiency of organic solvents in extracting bioactive secondary metabolites, such as fatty acids and phenolic compounds [43,44]. In addition to polarity-driven differences, Component 2 revealed a clear separation between ethanol and methanol extracts, indicating solvent-specific selectivity even among organic solvents. This suggests that methanol and ethanol differ in their ability to extract specific metabolite classes, likely due to subtle variations in polarity and hydrogen-bonding capacity [45]. The observed metabolomic differences have important implications for functional properties. From an applied perspective, these findings highlight the potential valorization of this invasive seaweed as a source of bioactive compounds. Moreover, the clear solvent-dependent differences indicate that targeted extraction strategies can be employed to optimize the recovery of specific metabolite classes. This approach aligns with current efforts to transform invasive marine biomass into valuable resources, thereby contributing to both environmental management and the development of biotechnological applications [46].

Total phenolic content determined using the Folin–Ciocalteu (F–C) reagent yielded similar values for the water and the ethanol extract, with no significant differences. This contrasts with the LC-QqQ results, which detected no phenolic compounds in the water extract. This discrepancy is not surprising, as it has been well documented that the F–C reagent reacts not only with phenolic compounds but also with proteins, thiols, vitamin derivatives, and certain inorganic ions. Consequently, the F–C assay should be interpreted as a measure of total antioxidant capacity rather than a specific quantification of phenolic content [47]. This was further confirmed by Luaces et al., who reported higher concentrations using the F–C method compared to HPLC, concluding that the F–C assay estimates the total content of reducing compounds, both phenolic and non-phenolic, whereas HPLC quantifies only the major phenolic compounds [48].

Potential contributors include water-soluble antioxidant compounds commonly reported in seaweeds, such as amino acids, vitamins, polysaccharides, peptides, and mycosporine-like amino acids [49,50,51,52,53,54,55], although these were not specifically quantified in the present study. This interpretation may also explain the elevated total non-flavonoid values, as the F–C reagent is used in their determination. Indeed, the water extracts exhibited the highest antioxidant activity, as indicated by both the ABTS and DPPH assays. However, the metabolites responsible for this effect were not specifically identified. Although untargeted metabolomic profiling demonstrated a distinct enrichment of polar compounds in aqueous extracts, no direct link between individual metabolites and antioxidant activity was established. Accordingly, any structure–activity relationship inferred from the present results should be considered preliminary, and future studies employing targeted metabolite identification and bioactivity-guided approaches will be necessary to determine the compounds underlying the observed antioxidant effects.

The total flavonoid content was highest in the ethanol extract, followed by methanol, and lowest in the water extract. This is because ethanol and methanol are more effective solvents for flavonoids, and the method used to determine total flavonoids (AlCl_3_ reaction) is more selective, unlike the completely non-specific reducing agent method, such as the Folin–Ciocalteu assay [56].

ABTS and DPPH activities were highest in the water extract, followed by the ethanolic and methanolic extracts for ABTS, and the methanolic and ethanolic extracts for DPPH, whereas FRAP activity was greatest in the ethanolic extract, followed by the methanolic and water extracts. The observed differences in antioxidant activities among ABTS, DPPH, and FRAP assays reflect both assay mechanisms and solvent-dependent solubility of compounds.

Higher ABTS and DPPH activities observed in aqueous extracts indicate that hydrophilic metabolites play a major role in radical scavenging capacity. This aligns with the metabolomic profile, which shows an enrichment of polar compounds, particularly organic acids and amino acids. These metabolites can contribute to antioxidant activity through mechanisms such as hydrogen atom transfer, electron donation, and metal chelation, although their overall contribution is typically secondary to that of phenolic compounds [49,50,51]. In contrast, the higher FRAP values observed in the ethanolic extract reflect a greater reducing power, indicating an enrichment of compounds with strong electron-donating capacity—most likely phenolic and other semi-polar constituents. Phenolic compounds are widely recognized for their redox properties and function as efficient reducing agents in ferric-reducing assays [1,47]. These findings highlight the mechanism-dependent nature of antioxidant assays, where different classes of compounds contribute to distinct antioxidant responses. Consequently, solvent selection plays a crucial role in shaping the antioxidant profile of extracts, as it directly affects the extraction efficiency of specific metabolite groups with diverse functional properties [9,57].

Similarly, Ouahabi et al. reported higher antioxidant activity in aqueous extracts of *C. prolifera* compared to organic extracts [31].

The observed correlation structure in PCA analyses is consistent with trends widely reported in the antioxidant literature of green seaweeds [58,59]. The very strong positive association between FRAP and TF supports the well-established role of flavonoids as major contributors to reducing power, as both assays are based on electron-donating capacity. The moderate correlation with TPC further confirms that phenolics collectively contribute to redox potential, although not all phenolics exhibit equal reducing efficiency [13]. Similar patterns have been described where FRAP aligns more closely with specific subclasses, such as flavonoids, rather than total phenolics.

The clustering of TNF with DPPH and ABTS into a secondary component aligns with other studies of *Caulerpa* spp. [60] highlighting that nitrogen-containing or non-phenolic compounds can contribute to radical scavenging activity.

The present study provides an initial characterization of the metabolomic profile and antioxidant properties of *C. cylindracea* from the Northern Adriatic Sea. However, samples were collected from a single location during a single season, which limits the broader applicability of the findings. Therefore, future studies incorporating multiple locations, seasons, and biological replicates will be necessary to assess spatial and temporal variability, confirm metabolite annotations, and establish the biological relevance of the observed patterns.

## 4. Materials and Methods

### 4.1. Material Collection and Storage

**Figure 12:**
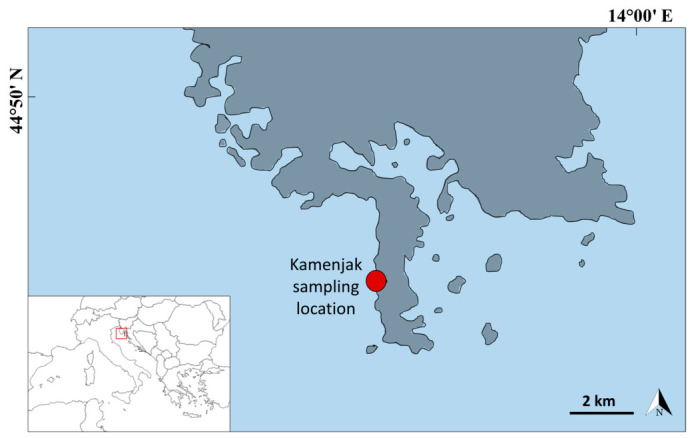
Sampling location in the Northern Adriatic Sea.

Samples of *C. cylindracea* were collected in July 2025 from the infralittoral area of the western coast of the Bay of Medulin in the proximity of the protected area Lower Kamenjak and Medulin archipelago at an average depth of 12 m (Figure 12). Sampling was conducted in the summer, since in the Mediterranean the production of algal secondary metabolites is seasonally regulated, with peak levels reported during vegetative growth (summer–autumn) [61]. Samples were morphologically identified as *C. cylindracea* Sonder and their taxonomic identity was confirmed by Dr. sc. Moira Buršić. The collected seaweed was soaked and afterwards washed thoroughly with running water to eliminate epibionts and sediment. The seaweed was dried in a food dehydrator (manufactured by Gorenje, Velenje, Slovenia, model FDK24DW) at 35 °C for 18 h. Afterwards, they were stored in a vacuum container in the dark until use.

### 4.2. Antioxidant Activity, Total Phenolic, Flavonoid, and Non-Flavonoid Content

The dried seaweed was ground into a fine powder. Extracts were prepared using the maceration method by dissolving 1 g of *C. cylindracea* powder in 10 mL of solvent (1:10 *w*/*v*) as described by Palayinappan et al. [62]. The mixtures were kept on a shaker for 24 h at room temperature [62], after which the extracts were centrifuged. The solvents used for extraction were water, 70% ethanol, and 80% methanol. Triplicate extracts were prepared for each solvent (N = 3). Each sample measurements were performed in triplicate.

The method developed by Singleton and Rossi [63] was used to determine total phenolics (TP), whereas total non-flavonoids (TNF) were measured following the protocol outlined by Ough and Amerine [64]. Both methods are based on the reduction of the Folin-Ciocalteu (FC) reagent by phenolic compounds, leading to the formation of a molybdenum-tungsten blue complex, which is subsequently measured spectrophotometrically at 765 nm. For the TP assay, 0.1 mL of distilled water and 0.1 mL of FC reagent were added. The mixture was left to react for 5 min at room temperature. Then, 1.5 mL of 20% sodium carbonate solution was added, and the reaction was allowed to develop for 30 min at room temperature (RT). The molybdenum-tungsten blue complex formation was measured spectrophotometrically at 765 nm. In the TNF assay, 0.1 mL of the extract was mixed with 1.5 mL of distilled water and 0.1 mL of FC reagent. The reaction was allowed to proceed for 5 min at room temperature. Subsequently, 1.5 mL of 20% sodium carbonate solution was added, and the mixture was incubated for 30 min at RT. The resulting blue complex was quantified spectrophotometrically at 765 nm. Results are expressed as mg of gallic acid equivalents (GAE) per gram of dry weight (DW).

Total flavonoid (TF) content was determined following the protocol described by Martins et al. [65]. In the TF assay, 1 mL of 2% aluminum chloride solution was added to 1 mL of the extract. After a 15-min incubation at room temperature (RT), the absorbance of the reaction mixture was measured spectrophotometrically at 430 nm. The results are expressed as mg of (+)-catechin equivalents (CE) per g of DW.

The antioxidant capacity (AC) of the extracts was determined spectrophotometrically using standard DPPH, ABTS, and FRAP assays, following the methods described by Poljuha et al. [66].

The DPPH assay was performed by mixing 0.1 mL of the extract with 3.9 mL of DPPH radical solution (0.1 mM in methanol). Following 30 min of incubation in the dark at room temperature, the absorbance of the mixture was measured at 517 nm. The antioxidant activity was calculated using a Trolox calibration curve and expressed as milligrams of Trolox equivalents per gram of dry weight (mg TE/g DW).

The FRAP assay was performed by mixing 0.1 mL of the extract with 3 mL of FRAP reagent, which consisted of 19 mM 2,4,6-tripyridyl-s-triazine in 40 mM HCl, 20 mM FeCl_3_, and 300 mM acetate buffer (pH 3.6). The mixture was incubated at room temperature for 30 min, after which the absorbance was measured at 593 nm. FRAP values were calculated using a Trolox calibration curve and expressed as milligrams of Trolox equivalents per gram of dry weight (mg TE/g DW).

For the ABTS assay, the ABTS^+^• radical cation was generated by reacting 7 mM ABTS solution with 2.45 mM potassium persulfate, followed by incubation in the dark at room temperature for 12 h. The resulting radical cation solution was then diluted with ethanol to achieve an absorbance of 0.70 ± 0.02 at 743 nm. A 100 µL aliquot of the extract was mixed with 3.9 mL of the ABTS solution, and after 6 min of reaction, the absorbance was measured at 734 nm. ABTS values were calculated using a Trolox calibration curve and expressed as milligrams of Trolox equivalents per gram of dry weight (mg TE/g DW).

All measurements were conducted in triplicate using a NanoPhotometer P300 spectrophotometer (Implen GmbH, Munich, Germany), calibrated for a 2 mL cuvette volume.

### 4.3. Chemical Composition of C. cylindracea

For extraction, 250 mg of dried material was homogenized in 10 mL of water, 70% aqueous ethanol and 80% aqueous methanol by vortex mixing (Biosan V-1, Riga, Latvia) for 2 min, followed by maceration on a rotary mixer (Biosan RS-60, Riga, Latvia) for 20 h at 25 °C. The resulting extracts were centrifuged at 4000× *g* for 10 min (Tehtnica Centric 350, Podplat, Slovenia) and filtered through a 0.22 μm nylon membrane filter before targeted and untargeted LC-MS/MS analysis.

Targeted polyphenolic compound identification and quantification was carried out using authentic reference standards by an LC-QqQ system comprising a system controller (SCL-40, Kyoto, Japan), degasser (DGU-405, Kyoto, Japan), two binary solvent delivery units (LC-40DX3, Kyoto, Japan), autosampler (SIL-40CX3, Kyoto, Japan), thermostated column compartment (CTO-40C, Kyoto, Japan), and a triple quadrupole mass spectrometer (LCMS-8045, Kyoto, Japan). Chromatographic separation was performed on a C18 core-shell column (150 mm × 2.1 mm, 2.7 μm; Agilent, Palo Alto, CA, USA) held at 37 °C, following the method of Major et al. [67]. A 3 μL injection volume was used, with separation achieved via binary gradient elution at 0.35 mL/min using water with 0.1% acetic acid (mobile phase A) and methanol with 0.1% acetic acid (mobile phase B). The gradient programme was as follows: 0–0.75 min, 95% A; 0.75–15 min, 95% A to 50% A; 15–15.1 min, 50% A to 0% A; 15.1–20 min, 0% A; 20–20.1 min, 0% A to 95% A; 20.1–25 min, 95% A.

Untargeted metabolomic analysis was performed on a Shimadzu LC-qTOF system comprising a system controller (Shimadzu CBM-40, Kyoto, Japan), solvent delivery module (Shimadzu LC-40D, Kyoto, Japan), degassing unit (Shimadzu DGU-405, Kyoto, Japan), autosampler (Shimadzu SIL-40X3, Kyoto, Japan), and column oven (Shimadzu CTO-40C, Kyoto, Japan), coupled to a qTOF mass spectrometer (Shimadzu LCMS-9050, Kyoto, Japan). The same extracts prepared for targeted analysis were used for untargeted profiling. Before injection, samples were diluted 1:1 with the initial mobile phase composition. Pooled quality control (QC) samples were prepared by combining equal volumes from all nine samples and were injected at the beginning and end of the analytical sequence to monitor instrument stability and for intensity normalization.

Chromatographic separation was achieved by injecting 3 µL of extract onto a 150 mm × 2.1 mm HALO biphenyl column (2.7 µm particle size; Advanced Materials Technology, Wilmington, DE, USA) maintained at 37 °C at a flow rate of 0.35 mL/min. Mobile phase A consisted of water with 0.1% acetic acid and mobile phase B of methanol with 0.1% acetic acid. The gradient elution was as follows: 0–1 min, 95% A; 1–10 min, 95% A to 0% A; 10–15 min, 0% A (hold); 15–15.1 min, 0% A to 95% A; 15.1–20 min, 95% A (re-equilibration).

MS detection was performed in Data Independent Acquisition (DIA) mode with a fixed isolation window of 20 *m*/*z* across a precursor range of 70–900 *m*/*z*, and MS2 fragments acquired over 40–900 *m*/*z*. Analyses were conducted in positive (ESI+) and negative (ESI−) ionization modes in separate runs. The ESI source voltage was set to +4.50 kV and −3.50 kV for positive and negative modes, respectively. Collision energy was applied as a ramp from 10 to 50 eV for MS2 fragmentation. The nebulizing, heating, and drying gas flows were set to 3, 10, and 10 L/min, respectively, while the interface, desolvation line, and heat block temperatures were maintained at 150, 120, and 400 °C, respectively. Mass accuracy was maintained throughout each run by injection of the mass calibration solution via the sub-interface.

Raw data were processed using MS-DIAL (v4.24). MS1 and MS2 mass tolerances were set to 0.01 Da and 0.025 Da, respectively, with a minimum peak height threshold of 1000 counts. Adduct and isotope deduplication were applied during peak detection. Chromatographic alignment was performed using a retention time tolerance of 0.15 min and an MS1 tolerance of 0.015 Da, without retention time correction. Peak intensities were normalized against the pooled QC samples, and features with a blank-to-sample ratio exceeding 0.2 (blank average/sample maximum) were removed to eliminate background contaminants. Metabolite annotation was performed against MassBank (version 19) using a minimum reverse dot-product score threshold of 0.8. In positive mode, the adducts [M+H]^+^, [M+NH_4_]^+^, [M+Na]^+^, and [M+K]^+^ were searched, while in negative mode [M−H]^−^, [M+HCOO]^−^, and [M+CH_3_COO]^−^ were used. Annotations are reported at MSI confidence level 2 (spectral library match without reference standard confirmation).

### 4.4. Data Analyse

Statistical analyses of phenolic compounds, total phenolics, total flavonoids, total non-flavonoids, and antioxidative capacity were performed using Statistica 9.0. (StatSoft Inc, Tulsa, OK, USA). The dataset was tested for normality using the Shapiro-Wilk test (*p* > 0.05), and homogeneity of variance was assessed with Levene’s test (*p* > 0.05). The *t*-test was used to detect differences in phenolic compounds between the ethanolic and methanolic extracts. The Kruskal-Wallis test, followed by the Mann-Whitney post hoc test, was employed to determine differences in total phenolics, total flavonoids and total non-flavonoids. Antioxidant capacity data measured with ABTS, DPPH and FRAP tests were analyzed using a one-way ANOVA test with post hoc Tukey HSD test.

Normalized peak intensities of polyphenolic compounds were imported into MetaboAnalyst 6.0 for statistical analysis. Data was log-transformed and auto-scaled prior to multivariate analysis. A sparse Partial Least Squares Discriminant Analysis (sPLS-DA) was applied to discriminate between the water, ethanolic, and methanolic extracts, with the final model retaining two components and ten variables per component, selected based on the lowest balanced error rate from 10-fold cross-validation. Features reaching statistical significance by one-way ANOVA (*p* < 0.05) were used for Hierarchical Clustering Analysis (HCA), visualized as a heatmap using Euclidean distance and the Ward.D linkage algorithm.

## 5. Conclusions

This study provides the first integrated characterization of the antioxidant properties, phenolic composition, and metabolomic profile of *C. cylindracea* from the Northern Adriatic Sea. Antioxidant activity varied among extraction solvents: aqueous extracts exhibited the highest ABTS and DPPH radical-scavenging activity, whereas ethanolic extracts showed the greatest ferric reducing capacity and flavonoid content.

Targeted LC-QqQ analysis identified seven phenolic compounds in ethanolic and methanolic extracts, with 4-hydroxybenzoic acid as the predominant constituent. Untargeted LC-qTOF metabolomics revealed a clear solvent-dependent extraction pattern Overall, the results highlight the chemical diversity of *C. cylindracea* and demonstrate the importance of solvent selection in shaping both metabolite composition and antioxidant properties. These findings support the potential valorization of this invasive seaweed as a source of bioactive compounds and provide a foundation for future research. As this study was based on samples collected from a single location during a single season, further investigations incorporating broader spatial and temporal sampling, biological replication, targeted metabolite confirmation, and functional validation are needed to assess the consistency and biological significance of the observed patterns.

## Figures and Tables

**Table 1:** Results of Multiple Comparisons *p* values (2-tailed) for total phenolic content (TPC), total flavonoids (TF) and total non-flavonoids (TNF).

Content	Kruskal-Wallis Test	H	*p*
TPC	2, N = 27	18.34	0.0001
TF	2, N = 27	23.15	0.0001
TNF	2, N = 27	19.70	0.0001

**Table 2:** Results of Multiple Comparisons *p* values (2-tailed) for total antioxidative capacity measured by ABTS, FRAP and DPPH.

Antioxidative Test	SS	df	MS	F	*p*
ABTS	1.40	2	0.70	8.85	0.0013
FRAP	1.28	2	0.64	365.38	0.0001
DPPH	0.54	2	0.27	7.10	0.0037

**Table 3:** Phenolic compounds found in *C. cylindracea* expressed in μg/kg DW. (The asterisks indicate a statistically significant difference calculated using the *t*-test: * *p* < 0.05; ** *p* < 0.001).

Compound	Retention Time	MRM	Water	Ethanol 70%	Methanol 80%
4-hydroxybenzoic acid	7.465	137.0 > 93.0	-	2742 ± 167	2696 ± 53
2-hydroxybenzoic acid	11.655	137.1 > 93.0	-	411.8 ± 88.5	504.8 ± 49.2
Vanillin	10.021	151.1 > 135.9	-	196 ± 19.7	229.3 ± 29.4
Scoparone	13.350	207.1 > 151.0	-	149.7 ± 23.7	129.75 ± 16.2
Diosgenin	24.211	415.3 > 271.1	-	44 ± 2.2	44.2 ± 2.7
Sarsasapogenin	24.385	417.3 > 273.2	-	41.9 ± 2.9	53.7 ± 8.7 *
Luteolin-4′-O-glucoside	15.302	447.1 > 285.0	-	14 ± 4.1	29.8 ± 3.5 **

## Data Availability

The original contributions presented in this study are included in the article/Supplementary Materials. Further inquiries can be directed to the corresponding author.

## Supplementary Materials

The following supporting information can be downloaded at: https://www.mdpi.com/article/10.3390/ph19071065/s1, Table S1: Annotated metabolites identified in *Caulerpa* extracts by LC–qTOF analysis.

## References

[1] Cotas J., Leandro A., Monteiro P., Pacheco D., Figueirinha A., Gonçalves A.M.M., da Silva G.J., Pereira L. (2020). Seaweed Phenolics: From Extraction to Applications. Mar. Drugs.

[2] Belton G.S., Draisma S.G.A., Prud’homme van Reine W.F., Huisman J.M., Gurgel C.F.D. (2019). A Taxonomic Reassessment of *Caulerpa* (Chlorophyta, Caulerpaceae) in Southern Australia, Based on tufA and rbcL Sequence Data. Phycologia.

[3] Stabili L., Fraschetti S., Acquaviva M.I., Cavallo R.A., De Pascali S.A., Fanizzi F.P., Gerardi C., Narracci M., Rizzo L. (2016). The Potential Exploitation of the Mediterranean Invasive Alga *Caulerpa cylindracea*: Can the Invasion Be Transformed into a Gain?. Mar. Drugs.

[4] Najdek M., Korlević M., Paliaga P., Markovski M., Ivančić I., Iveša L., Felja I., Herndl G.J. (2020). Effects of the Invasion of *Caulerpa cylindracea* in a *Cymodocea nodosa* Meadow in the Northern Adriatic Sea. Front. Mar. Sci..

[5] Gomez-Zavaglia A., Prieto Lage M.A., Jimenez-Lopez C., Mejuto J.C., Simal-Gandara J. (2019). The Potential of Seaweeds as a Source of Functional Ingredients of Prebiotic and Antioxidant Value. Antioxidants.

[6] Rotter A., Klun K., Francé J., Mozetič P., Orlando-Bonaca M. (2020). Non-Indigenous Species in the Mediterranean Sea: Turning from Pest to Source by Developing the 8Rs Model, a New Paradigm in Pollution Mitigation. Front. Mar. Sci..

[7] Tsiamis K., Azzurro E., Bariche M., Çinar M.E., Crocetta F., De Clerck O., Galil B., Gómez F., Hoffman R., Jensen K.R. (2020). Prioritizing Marine Invasive Alien Species in the European Union through Horizon Scanning. Aquat. Conserv. Mar. Freshw. Ecosyst..

[8] Giakoumi S., Katsanevakis S., Albano P.G., Azzurro E., Cardoso A.C., Cebrian E., Deidun A., Edelist D., Francour P., Jimenez C. (2019). Management Priorities for Marine Invasive Species. Sci. Total Environ..

[9] Amin I., Tan S.H. (2002). Antioxidant Activity of Selected Commercial Seaweeds. Malays. J. Nutr..

[10] Kohen R., Nyska A. (2002). Invited Review: Oxidation of Biological Systems: Oxidative Stress Phenomena, Antioxidants, Redox Reactions, and Methods for Their Quantification. Toxicol. Pathol..

[11] Brix da Costa B., Kunzmann A., Springer K. (2025). Comparative Analysis of the Nutritional Profiles of Five Edible Macroalgae as Sustainable Food Sources. Discov. Food.

[12] Iveša N., Kovačić I., Buršić M., Major N., Palčić I., Goreta Ban S., Užila Z., Millotti G. (2025). *Caulerpa cylindracea*: First Insight into Its Nutritional Potential. Foods.

[13] Tahar A., Zghida H., Pereira D.T., Korbee N., Treichel H., Figueroa F.L., Achour L. (2025). Biochemical Composition and Alkaline Extraction Optimization of Soluble Bioactive Compounds from the Green Algae *Caulerpa cylindraceae*. Mar. Drugs.

[14] Erol E., Didem Orhan M., Avsar T., Akdemir A., Sukran Okudan E., Toraman G.O.A., Topcu G. (2022). Anti-SARS-CoV-2 and Cytotoxic Activity of Two Marine Alkaloids from Green Alga *Caulerpa cylindracea* Sonder in the Dardanelles. RSC Adv..

[15] Belkacemi L., Belalia M., Djendara A.C., Bouhadda Y. (2020). Antioxidant and Antibacterial Activities and Identification of Bioactive Compounds of Various Extracts of *Caulerpa racemosa* from Algerian Coast. Asian Pac. J. Trop. Biomed..

[16] Alara O.R., Abdurahman N.H., Ukaegbu C.I. (2021). Extraction of Phenolic Compounds: A Review. Curr. Res. Food Sci..

[17] Božac M.U., Poljuha D., Dudaš S., Bilić J., Šola I., Mikulič-Petkovšek M., Sladonja B. (2025). Phytochemical Profile and Antioxidant Properties of Invasive Plants *Ailanthus altissima* (Mill.) Swingle and *Helianthus tuberosus* L. in Istria Region, Croatia. Antioxidants.

[18] Babbar N., Oberoi H.S., Sandhu S.K., Bhargav V.K. (2014). Influence of Different Solvents in Extraction of Phenolic Compounds from Vegetable Residues and Their Evaluation as Natural Sources of Antioxidants. J. Food Sci. Technol..

[19] Yang J., Ou X., Zhang X., Zhou Z., Ma L. (2017). Effect of Different Solvents on the Measurement of Phenolics and the Antioxidant Activity of Mulberry (*Morus atropurpurea* Roxb.) with Accelerated Solvent Extraction. J. Food Sci..

[20] Rosiles-Alanis W., Zamilpa A., García-Macedo R., Zavala-Sánchez M.A., Hidalgo-Figueroa S., Mora-Ramiro B., Román-Ramos R., Estrada-Soto S.E., Almanza-Perez J.C. (2022). 4-Hydroxybenzoic Acid and β-Sitosterol from *Cucurbita ficifolia* Act as Insulin Secretagogues, Peroxisome Proliferator-Activated Receptor-Gamma Agonists, and Liver Glycogen Storage Promoters: In Vivo, In Vitro, and In Silico Studies. J. Med. Food.

[21] Peungvicha P., Thirawarapan S.S., Watanabe H. (1998). Possible Mechanism of Hypoglycemic Effect of 4-Hydroxybenzoic Acid, a Constituent of *Pandanus Odorus* Root. Jpn. J. Pharmacol..

[22] Kosová M., Hrádková I., Mátlová V., Kadlec D., Šmidrkal J., Filip V. (2015). Antimicrobial Effect of 4-Hydroxybenzoic Acid Ester with Glycerol. J. Clin. Pharm. Ther..

[23] Mahdi J., Al-Musayeib N., Mahdi E., Pepper C. (2013). Pharmacological Importance of Simple Phenolic Compounds on Inflammation, Cell Proliferation and Apoptosis with a Special Reference to β-D-Salicin and Hydroxybenzoic Acid. Eur. J. Inflamm..

[24] Choi Y.H., Yan G.H. (2009). Anti-Allergic Effects of Scoparone on Mast Cell-Mediated Allergy Model. Phytomedicine.

[25] Li N., Yang F., Liu D.-Y., Guo J.-T., Ge N., Sun S.-Y. (2021). Scoparone Inhibits Pancreatic Cancer through PI3K/Akt Signaling Pathway. World J. Gastrointest. Oncol..

[26] Hui Y., Wang X., Yu Z., Fan X., Cui B., Zhao T., Mao L., Feng H., Lin L., Yu Q. (2020). Scoparone as a Therapeutic Drug in Liver Diseases: Pharmacology, Pharmacokinetics and Molecular Mechanisms of Action. Pharmacol. Res..

[27] Arya S.S., Rookes J.E., Cahill D.M., Lenka S.K. (2021). Vanillin: A Review on the Therapeutic Prospects of a Popular Flavouring Molecule. Adv. Tradit. Med..

[28] Semwal P., Painuli S., Abu-Izneid T., Rauf A., Sharma A., Daştan S.D., Kumar M., Alshehri M.M., Taheri Y., Das R. (2022). Diosgenin: An Updated Pharmacological Review and Therapeutic Perspectives. Oxidative Med. Cell. Longev..

[29] Mustafa N.H., Sekar M., Fuloria S., Begum M.Y., Gan S.H., Rani N.N.I.M., Ravi S., Chidambaram K., Subramaniyan V., Sathasivam K.V. (2022). Chemistry, Biosynthesis and Pharmacology of Sarsasapogenin: A Potential Natural Steroid Molecule for New Drug Design, Development and Therapy. Molecules.

[30] Lin Y., Liu P.-G., Liang W.-Q., Hu Y.-J., Xu P., Zhou J., Pu J.-B., Zhang H.-J. (2018). Luteolin-4′-*O*-Glucoside and Its Aglycone, Two Major Flavones of *Gnaphalium affine* D. Don, Resist Hyperuricemia and Acute Gouty Arthritis Activity in Animal Models. Phytomedicine.

[31] Ouahabi S., Daoudi N.E., Chebaibi M., Mssillou I., Rahhou I., Bnouham M., Hammouti B., Fauconnier M.-L., Ayerdi Gotor A., Rhazi L. (2025). A Comparative Study of the Phytochemical Composition, Antioxidant Properties, and In Vitro Anti-Diabetic Efficacy of Different Extracts of *Caulerpa prolifera*. Mar. Drugs.

[32] Zhong B., Robinson N.A., Warner R.D., Barrow C.J., Dunshea F.R., Suleria H.A.R. (2020). LC-ESI-QTOF-MS/MS Characterization of Seaweed Phenolics and Their Antioxidant Potential. Mar. Drugs.

[33] Biełach-Bazyluk A., Jakubowicz-Zalewska O., Myśliwiec H., Flisiak I. (2025). Specialized Pro-Resolving Lipid Mediators and Dietary Omega-3/6 Fatty Acids in Selected Inflammatory Skin Diseases: A Systematic Review. Antioxidants.

[34] Dalli J., Winkler J.W., Colas R.A., Arnardottir H., Cheng C.-Y.C., Chiang N., Petasis N.A., Serhan C.N. (2013). Resolvin D3 and Aspirin-Triggered Resolvin D3 Are Potent Immunoresolvents. Chem. Biol..

[35] Hellmann J., Sansbury B.E., Wong B., Li X., Singh M., Nuutila K., Chiang N., Eriksson E., Serhan C.N., Spite M. (2018). Biosynthesis of D-Series Resolvins in Skin Provides Insights into Their Role in Tissue Repair. J. Investig. Dermatol..

[36] Isobe Y., Arita M., Matsueda S., Iwamoto R., Fujihara T., Nakanishi H., Taguchi R., Masuda K., Sasaki K., Urabe D. (2012). Identification and Structure Determination of Novel Anti-Inflammatory Mediator Resolvin E3, 17,18-Dihydroxyeicosapentaenoic Acid*. J. Biol. Chem..

[37] Galanty A., Grudzińska M., Paździora W., Paśko P. (2023). Erucic Acid—Both Sides of the Story: A Concise Review on Its Beneficial and Toxic Properties. Molecules.

[38] Schild J., Kalvodová A., Zbytovská J., Farwick M., Pyko C. (2024). The Role of Ceramides in Skin Barrier Function and the Importance of Their Correct Formulation for Skincare Applications. Int. J. Cosmet. Sci..

[39] Wakeel A., Jan S.A., Ullah I., Shinwari Z.K., Xu M. (2019). Solvent Polarity Mediates Phytochemical Yield and Antioxidant Capacity of Isatis Tinctoria. PeerJ.

[40] Ao J., Li B. (2012). Amino Acid Composition and Antioxidant Activities of Hydrolysates and Peptide Fractions from Porcine Collagen. Food Sci. Technol. Int..

[41] Carocho M., Ferreira I.C.F.R. (2013). A Review on Antioxidants, Prooxidants and Related Controversy: Natural and Synthetic Compounds, Screening and Analysis Methodologies and Future Perspectives. Food Chem. Toxicol..

[42] Fratianni F., d’Acierno A., Ombra M.N., Amato G., De Feo V., Ayala-Zavala J.F., Coppola R., Nazzaro F. (2021). Fatty Acid Composition, Antioxidant, and in Vitro Anti-Inflammatory Activity of Five Cold-Pressed Prunus Seed Oils, and Their Anti-Biofilm Effect Against Pathogenic Bacteria. Front. Nutr..

[43] Cikoš A.-M., Jokić S., Šubarić D., Jerković I. (2018). Overview on the Application of Modern Methods for the Extraction of Bioactive Compounds from Marine Macroalgae. Mar. Drugs.

[44] Holdt S.L., Kraan S. (2011). Bioactive Compounds in Seaweed: Functional Food Applications and Legislation. J. Appl. Phycol..

[45] Lee J.-E., Jayakody J.T.M., Kim J.-I., Jeong J.-W., Choi K.-M., Kim T.-S., Seo C., Azimi I., Hyun J., Ryu B. (2024). The Influence of Solvent Choice on the Extraction of Bioactive Compounds from Asteraceae: A Comparative Review. Foods.

[46] Matos M., Custódio L., Reis C.P. (2024). Marine Invasive Algae’s Bioactive Ingredients as a Sustainable Pathway in Cosmetics: The Azores Islands as a Case Study. Mar. Drugs.

[47] Everette J.D., Bryant Q.M., Green A.M., Abbey Y.A., Wangila G.W., Walker R.B. (2010). Thorough Study of Reactivity of Various Compound Classes toward the Folin–Ciocalteu Reagent. J. Agric. Food Chem..

[48] Luaces P., Pascual M., Pérez A.G., Sanz C. (2021). An Easy-to-Use Procedure for the Measurement of Total Phenolic Compounds in Olive Fruit. Antioxidants.

[49] Xu N., Chen G., Liu H. (2017). Antioxidative Categorization of Twenty Amino Acids Based on Experimental Evaluation. Molecules.

[50] Michalak I., Tiwari R., Dhawan M., Alagawany M., Farag M.R., Sharun K., Emran T.B., Dhama K. (2022). Antioxidant Effects of Seaweeds and Their Active Compounds on Animal Health and Production—A Review. Vet. Q..

[51] Charlton N.C., Mastyugin M., Török B., Török M. (2023). Structural Features of Small Molecule Antioxidants and Strategic Modifications to Improve Potential Bioactivity. Molecules.

[52] Arunkumar K., Raja R., Kumar V.B.S., Joseph A., Shilpa T., Carvalho I.S. (2021). Antioxidant and Cytotoxic Activities of Sulfated Polysaccharides from Five Different Edible Seaweeds. J. Food Meas. Charact..

[53] Echave J., Otero P., Garcia-Oliveira P., Munekata P.E.S., Pateiro M., Lorenzo J.M., Simal-Gandara J., Prieto M.A. (2022). Seaweed-Derived Proteins and Peptides: Promising Marine Bioactives. Antioxidants.

[54] Li W., Su H.-N., Pu Y., Chen J., Liu L.-N., Liu Q., Qin S. (2019). Phycobiliproteins: Molecular Structure, Production, Applications, and Prospects. Biotechnol. Adv..

[55] Geraldes V., Pinto E. (2021). Mycosporine-Like Amino Acids (MAAs): Biology, Chemistry and Identification Features. Pharmaceuticals.

[56] Pękal A., Pyrzynska K. (2014). Evaluation of Aluminium Complexation Reaction for Flavonoid Content Assay. Food Anal. Methods.

[57] El Mannoubi I. (2023). Impact of Different Solvents on Extraction Yield, Phenolic Composition, in Vitro Antioxidant and Antibacterial Activities of Deseeded Opuntia Stricta Fruit. J. Umm Al-Qura Univ. Appl. Sci..

[58] Tanna B., Choudhary B., Mishra A. (2018). Metabolite Profiling, Antioxidant, Scavenging and Anti-Proliferative Activities of Selected Tropical Green Seaweeds Reveal the Nutraceutical Potential of *Caulerpa* spp.. Algal Res..

[59] Matanjun P., Mohamed S., Noordin M.M., Muhammad K., Ming C. (2008). Antioxidant Activities and Phenolics Content of Eight Species of Seaweeds from North Borneo. J. Appl. Phycol..

[60] Yasman Y., Akhmad A. (2025). Antioxidant Bioactivity of *Caulerpa* spp.: Potential, Challenges, and Future Research Directions. Egypt. J. Phycol..

[61] Box A., Deudero S., Sureda A., Blanco A., Alòs J., Terrados J., Grau A.M., Riera F. (2009). Diet and Physiological Responses of *Spondyliosoma cantharus* (Linnaeus, 1758) to the *Caulerpa racemosa* Var. *Cylindracea* Invasion. J. Exp. Mar. Biol. Ecol..

[62] Palaniyappan S., Sridhar A., Kari Z.A., Téllez-Isaías G., Ramasamy T. (2023). Evaluation of Phytochemical Screening, Pigment Content, In Vitro Antioxidant, Antibacterial Potential and GC-MS Metabolite Profiling of Green Seaweed *Caulerpa racemosa*. Mar. Drugs.

[63] Singleton V.L., Rossi J.A. (1965). Colorimetry of Total Phenolics with Phosphomolybdic-Phosphotungstic Acid Reagents. Am. J. Enol. Vitic..

[64] Ough C.S., Amerina M.A. (1988). Methods for Analysis of Musts and Wines.

[65] Martins D., Barros L., Carvalho A.M., Ferreira I.C.F.R. (2011). Nutritional and in Vitro Antioxidant Properties of Edible Wild Greens in Iberian Peninsula Traditional Diet. Food Chem..

[66] Poljuha D., Šola I., Bilić J., Dudaš S., Bilušić T., Markić J., Rusak G. (2015). Phenolic Composition, Antioxidant Capacity, Energy Content and Gastrointestinal Stability of Croatian Wild Edible Plants. Eur. Food Res. Technol..

[67] Major N., Išić N., Kovačević T.K., Anđelini M., Ban D., Prelac M., Palčić I., Ban S.G. (2023). Size Does Matter: The Influence of Bulb Size on the Phytochemical and Nutritional Profile of the Sweet Onion Landrace “Premanturska Kapula” (*Allium cepa* L.). Antioxidants.

