# Supplementary material for: Solvent-Dependent Metabolomic Profiles and Antioxidant Properties of the Invasive Seaweed Caulerpa cylindracea from the Adriatic Sea
Source: Pharmaceuticals (Basel). 2026 Jul 10;19(7):1065. doi: 10.3390/ph19071065 (PMC13415091; doi:10.3390/ph19071065)
Supplement: Supplementary file 1 [file pharmaceuticals-19-01065-s001.zip › pharmaceuticals-4384095-supplementary.pdf]

Table S1. Annotated metabolites identified in Caulerpa extracts by LC–qTOF analysis.

Alignment ID	Average Rt(min)	Average Mz	ESI	Compound Name
3	0,981	87,0078	neg	Pyruvic acid
63	0,935	148,0594	pos	L-Glutamic acid
67	1,234	150,0568	pos	Methionine
193	4,312	205,0962	pos	Tryptophan
96	2,608	166,0853	pos	Phenylalanine
133	1,247	182,0804	pos	Tyrosine
13	1,255	111,0189	neg	Uracil
15	1,598	115,0386	neg	2-Oxovaleric acid
12	0,994	104,1061	pos	Choline
37	1,27	132,1012	pos	Leucine
43	2,939	134,0459	neg	ADENINE
44	1,452	134,0459	neg	ADENINE
27	1,005	116,0701	pos	L-Proline
41	1,026	133,0132	neg	L-(-)-Malic acid
499	6,154	298,0948	pos	5'-S-Methylthioadenosine
24	2,378	125,0346	neg	Thymine
4	1,138	89,0234	neg	DL-Lactic acid
402	2,974	268,1025	pos	Adenosine
490	11,629	303,2317	neg	Arachidonic acid
491	11,827	303,2317	neg	Arachidonic acid
78	1,325	150,0409	neg	Guanine
31	1,674	124,0386	pos	Nicotinic acid
882	7,805	377,1439	pos	Riboflavin
11	0,971	105,0182	neg	D-(+)-Glyceric acid
47	1,534	137,0451	pos	Hypoxanthine
354	2,058	267,0723	neg	Inosine
560	0,809	317,0561	pos	Uridine
88	1,069	162,1112	pos	L-Carnitine
156	0,996	190,05	pos	Kynurenic acid
351	2,952	266,0886	neg	2'-Deoxyguanosine
1845	13,74	883,5375	neg	PI 38:5
80	1,572	151,0249	neg	Xanthine
1467	11,532	529,1502	pos	Guanosine
277	1,465	243,0609	neg	Pseudouridine
1702	13,229	743,4839	neg	PG 34:3
1703	13,385	743,4843	neg	PG 34:3
1710	13,276	745,501	neg	PG 34:2
1711	13,432	745,5013	neg	PG 34:2
628	9,489	351,2167	neg	PGI2
29	1,058	118,0855	pos	Betaine
48	5,094	137,0232	neg	Salicylic acid
23	1,663	122,0236	neg	Isonicotinic acid
511	10,421	302,3035	pos	Sphinganine (d18:0)
959	11,105	395,1151	pos	3-Hydroxycinnamic acid
87	0,951	162,0749	pos	D-2-Aminoadipic acid
58	1,129	146,0912	pos	4-GUANIDINOBUTANOATE
655	9,65	340,2798	pos	D-ribo-Phytosphingosine
516	10,984	303,2296	pos	Linoleic acid
517	11,538	303,23	pos	Linoleic acid

367	0,951	255,1063	pos	MELATONIN
32	1,459	128,0339	neg	Pyroglutamic acid
33	1,217	128,0341	neg	Pyroglutamic acid
1460	0,941	527,158	pos	Maltotriose
98	0,991	168,068	pos	4-ACETAMIDOBUTANOATE
30	2,61	120,0798	pos	PHENYLETHANOLAMINE
464	4,117	296,0986	neg	2-O-Methylguanosine
10	1,522	103,0389	neg	2-Hydroxyisobutyric acid
229	11,159	227,2004	neg	Myristic acid
383	11,456	277,216	neg	a-Linolenic acid
767	0,934	365,104	pos	Gentiobiose
595	12,052	337,3096	neg	ERUCIC ACID
307	11,371	253,2161	neg	Palmitoleic acid
1891	12,061	681,4104	pos	Fucoxanthin
458	10,802	295,2264	neg	9-HODE
565	11,246	317,2088	pos	13-OxoODE
564	10,689	317,2069	pos	13-OxoODE
575	11,86	331,2627	neg	Docosatetraenoic acid
599	12,11	339,3253	neg	Behenic acid
669	12,26	367,3566	neg	Lignoceric acid
363	11,589	269,2471	neg	HEPTADECANOATE
518	11,875	309,2784	neg	Eicosenoic acid
667	12,219	365,3412	neg	Nervonic acid
600	10,641	325,2365	pos	GLYCEROL-MYRISTATE
1623	12,142	696,6135	neg	Cer[AS] d40:1
1635	12,653	706,6343	neg	Cer[NS] d42:2
485	9,478	292,0941	pos	Acetamide derivative
1180	11,634	449,1622	pos	Glucopyranosyl-butyrate
444	10,609	280,1299	pos	Galactosyl-diglyceride
553	10,032	315,1553	pos	Caryophyllene-type sesquiterpene
701	9,046	351,2429	pos	Heptadecenyl acetate
422	9,3	273,1451	pos	Cycloheptene derivative
1505	11,072	537,3015	pos	Penten-ol derivative
569	9,61	318,2998	pos	Phytosphingosine (iso)
106	7,88	173,117	neg	FA 9:0+1O
107	1,052	174,0758	neg	C7H13NO4
445	11,125	291,1953	neg	FA 18:4+1O
42	1,219	133,0493	neg	Dihydroxy-Valerate
453	11,233	293,2106	neg	FA 18:2+O
454	10,687	293,211	neg	FA 18:2+O
521	10,427	311,2212	neg	FA 18:2+2O
564	9,011	327,2159	neg	Trihydroxyoctadeca-dienoic acid
500	4,126	298,1142	pos	3'-O-Methylguanosine
21	6,251	121,0285	neg	3-Methoxycatechol
22	7,515	121,0285	neg	3-Methoxycatechol
482	7,615	291,155	pos	Cyclohexyl butanoic acid deriv.
561	11,106	317,1088	pos	Aspartame-like dipeptide
515	10,861	309,2045	neg	Diterpenoid ester
689	10,686	375,2141	neg	Resolvin D3
533	10,828	317,2109	neg	15-HEPE

449	11,401	282,278	pos	Oleamide
581	10,498	320,2562	pos	Palmitoleoyl Ethanolamide
570	9,229	329,2322	neg	Trihydroxyoctadec-enoic acid
493	11,241	295,226	pos	Hydroxy octadecatrienoic
1043	7,635	415,2075	pos	Erucamide
166	1,274	193,0149	pos	Uracil acetic acid
44	6,069	136,0726	pos	3-Aminoacetophenon
448	0,841	292,8439	neg	3,5-Dibromo-4-hydroxybenzoic acid

Unique ID	Database ID
Pyruvic acid_87.0078_0.981	[KEGG: C00022]
L-Glutamic acid_148.0594_0.935	[KEGG: C00025]
Methionine_150.0568_1.234	[KEGG: C00073]
Tryptophan_205.0962_4.312	[KEGG: C00078]
Phenylalanine_166.0853_2.608	[KEGG: C00079]
Tyrosine_182.0804_1.247	[KEGG: C00082]
Uracil_111.0189_1.255	[KEGG: C00106]
2-Oxovaleric acid_115.0386_1.598	[KEGG: C00109]
Choline_104.1061_0.994	[KEGG: C00114]
Leucine_132.1012_1.27	[KEGG: C00123]
ADENINE_134.0459_2.939	[KEGG: C00147]
ADENINE_134.0459_1.452	[KEGG: C00147]
L-Proline_116.0701_1.005	[KEGG: C00148]
L-(-)-Malic acid_133.0132_1.026	[KEGG: C00149]
5'-S-Methylthioadenosine_298.0948_6.154	[KEGG: C00170]
Thymine_125.0346_2.378	[KEGG: C00178]
DL-Lactic acid_89.0234_1.138	[KEGG: C00186]
Adenosine_268.1025_2.974	[KEGG: C00212]
Arachidonic acid_303.2317_11.629	[KEGG: C00219]
Arachidonic acid_303.2317_11.827	[KEGG: C00219]
Guanine_150.0409_1.325	[KEGG: C00242]
Nicotinic acid_124.0386_1.674	[KEGG: C00253]
Riboflavin_377.1439_7.805	[KEGG: C00255]
D-(+)-Glyceric acid_105.0182_0.971	[KEGG: C00258]
Hypoxanthine_137.0451_1.534	[KEGG: C00262]
Inosine_267.0723_2.058	[KEGG: C00294]
Uridine_317.0561_0.809	[KEGG: C00299]
L-Carnitine_162.1112_1.069	[KEGG: C00318]
Kynurenic acid_190.05_0.996	[KEGG: C00328]
2'-Deoxyguanosine_266.0886_2.952	[KEGG: C00330]
PI 38:5_883.5375_13.74	[KEGG: C00350 (class)]
Xanthine_151.0249_1.572	[KEGG: C00385]
Guanosine_529.1502_11.532	[KEGG: C00387]
Pseudouridine_243.0609_1.465	[KEGG: C00566]
PG 34:3_743.4839_13.229	[KEGG: C00644 (class)]
PG 34:3_743.4843_13.385	[KEGG: C00644 (class)]
PG 34:2_745.501_13.276	[KEGG: C00644 (class)]
PG 34:2_745.5013_13.432	[KEGG: C00644 (class)]
PGI2_351.2167_9.489	[KEGG: C00696]
Betaine_118.0855_1.058	[KEGG: C00719]
Salicylic acid_137.0232_5.094	[KEGG: C00805]
Isonicotinic acid_122.0236_1.663	[KEGG: C00833]
Sphinganine (d18:0)_302.3035_10.421	[KEGG: C00835]
3-Hydroxycinnamic acid_395.1151_11.105	[KEGG: C00849]
D-2-Aminoadipic acid_162.0749_0.951	[KEGG: C00956]
4-GUANIDINOBUTANOATE_146.0912_1.129	[KEGG: C01035]
D-ribo-Phytosphingosine_340.2798_9.65	[KEGG: C01114]
Linoleic acid_303.2296_10.984	[KEGG: C01595]
Linoleic acid_303.23_11.538	[KEGG: C01595]

MELATONIN_255.1063_0.951	[KEGG: C01598]
Pyroglutamic acid_128.0339_1.459	[KEGG: C01879]
Pyroglutamic acid_128.0341_1.217	[KEGG: C01879]
Maltotriose_527.158_0.941	[KEGG: C01935]
4-ACETAMIDOBUTANOATE_168.068_0.991	[KEGG: C02102]
PHENYLETHANOLAMINE_120.0798_2.61	[KEGG: C05335]
2-O-Methylguanosine_296.0986_4.117	[KEGG: C05417]
2-Hydroxyisobutyric acid_103.0389_1.522	[KEGG: C06082]
Myristic acid_227.2004_11.159	[KEGG: C06424]
a-Linolenic acid_277.216_11.456	[KEGG: C06427]
Gentiobiose_365.104_0.934	[KEGG: C08234]
ERUCIC ACID_337.3096_12.052	[KEGG: C08323]
Palmitoleic acid_253.2161_11.371	[KEGG: C08362]
Fucoxanthin_681.4104_12.061	[KEGG: C08591]
9-HODE_295.2264_10.802	[KEGG: C14825]
13-OxoODE_317.2088_11.246	[KEGG: C14828]
13-OxoODE_317.2069_10.689	[KEGG: C14828]
Docosatetraenoic acid_331.2627_11.86	[KEGG: C16527]
Behenic acid_339.3253_12.11	[KEGG: C16538]
Lignoceric acid_367.3566_12.26	[KEGG: C16539]
HEPTADECANOATE_269.2471_11.589	[KEGG: C16571]
Eicosenoic acid_309.2784_11.875	[KEGG: C16577]
Nervonic acid_365.3412_12.219	[KEGG: C16584]
GLYCEROL-MYRISTATE_325.2365_10.641	[KEGG: C19329]
Cer[AS] d40:1_696.6135_12.142	[LIPID MAPS: LMSR01010001]
Cer[NS] d42:2_706.6343_12.653	[LIPID MAPS: LMSR01010010]
Acetamide derivative_292.0941_9.478	[NCGC: 00380372]
Glucopyranosyl-butyrate_449.1622_11.634	[NCGC: 00380647]
Galactosyl-diglyceride_280.1299_10.609	[NCGC: 00380867]
Caryophyllene-type sesquiterpene_315.1553_10.032	[NCGC: 00384680]
Heptadecenyl acetate_351.2429_9.046	[NCGC: 00385642]
Cycloheptene derivative_273.1451_9.3	[NCGC: 00385912]
Penten-ol derivative_537.3015_11.072	[NCGC: 00385919]
Phytosphingosine (iso)_318.2998_9.61	[PlaSMA: ID-1697]
FA 9:0+1O_173.117_7.88	[PlaSMA: ID-176]
C7H13NO4_174.0758_1.052	[PlaSMA: ID-178]
FA 18:4+1O_291.1953_11.125	[PlaSMA: ID-539]
Dihydroxy-Valerate_133.0493_1.219	[PlaSMA: ID-60]
FA 18:2+O_293.2106_11.233	[PlaSMA: ID-618]
FA 18:2+O_293.211_10.687	[PlaSMA: ID-618]
FA 18:2+2O_311.2212_10.427	[PlaSMA: ID-643]
Trihydroxyoctadeca-dienoic acid_327.2159_9.011	[PubChem: 101416955]
3'-O-Methylguanosine_298.1142_4.126	[PubChem: 111166]
3-Methoxycatechol_121.0285_6.251	[PubChem: 11467]
3-Methoxycatechol_121.0285_7.515	[PubChem: 11467]
Cyclohexyl butanoic acid deriv._291.155_7.615	[PubChem: 129312155]
Aspartame-like dipeptide_317.1088_11.106	[PubChem: 129320625]
Diterpenoid ester_309.2045_10.861	[PubChem: 20586419]
Resolvin D3_375.2141_10.686	[PubChem: 44251261]
15-HEPE_317.2109_10.828	[PubChem: 445217]

Oleamide_282.278_11.401	[PubChem: 5283305]
Palmitoleoyl Ethanolamide_320.2562_10.498	[PubChem: 5283446]
Trihydroxyoctadec-enoic acid_329.2322_9.229	[PubChem: 5312788]
Hydroxy octadecatrienoic_295.226_11.241	[PubChem: 5312814]
Erucamide_415.2075_7.635	[PubChem: 5365371]
Uracil acetic acid_193.0149_1.274	[PubChem: 73934]
3-Aminoacetophenon_136.0726_6.069	[PubChem: 7508]
3,5-Dibromo-4-hydroxybenzoic acid_292.8439_0.841	[PubChem: 75525]

Chemical Class / Description	Adduct type	Post curation result	Fill %
Alpha-keto acid (Primary metabolite)	[M-H2O-H]-	null	0,19
Amino acid	[M+H]+	null	0,3
Essential Amino Acid	[M+H]+	null	0,11
Amino Acid	[M+H]+	null	0,33
Amino Acid	[M+H]+	null	0,44
Amino Acid	[M+H]+	null	0,63
Pyrimidine nucleobase (RNA)	[M-H]-	null	0,63
Alpha-keto acid (Amino acid metabolism)	[M-H]-	null	0,11
Essential nutrient / Phospholipid precursor	[M]+	null	0,63
Amino acid	[M+H]+	null	0,59
Purine nucleobase (DNA/RNA/ATP)	[M-H]-	null	0,48
Purine nucleobase (DNA/RNA/ATP)	[M-H]-	null	0,48
Amino Acid	[M+H]+	null	0,52
Dicarboxylic acid (TCA cycle)	[M-H]-	null	0,15
Methionine/Salvage cycle metabolite	[M+H]+	null	0,37
Pyrimidine nucleobase (DNA)	[M-H]-	null	0,63
Hydroxy acid (Fermentation product)	[M-H]-	null	0,63
Nucleoside	[M+H]+	null	0,52
Omega-6 Fatty Acid (20:4)	[M-H]-	null	0,63
Omega-6 Fatty Acid (20:4)	[M-H]-	null	0,44
Purine nucleobase (DNA/RNA)	[M-H]-	null	0,52
Vitamin B3	[M+H]+	null	0,59
Vitamin B2	[M+H]+	null	0,48
Sugar acid (Photosynthesis intermediate)	[M-H]-	null	0,26
Purine base	[M+H]+	null	0,59
Purine nucleoside	[M-H]-	null	0,52
Nucleoside	[M+H]+	null	0,63
Fatty acid transporter	[M+H]+	null	0,56
Tryptophan metabolite	[M+H]+	null	0,63
Purine nucleoside (DNA component)	[M-H]-	null	0,52
Phosphatidylinositol (Membrane lipid)	[M-H]-	null	0,11
Purine derivative	[M-H]-	null	0,63
Nucleoside	[M+Na]+	null	0,22
Isomerized nucleoside (RNA component)	[M-H]-	null	0,52
Phosphatidylglycerol (Membrane lipid)	[M-H]-	null	0,19
(Duplicate entry)	[M-H]-	null	0,11
Phosphatidylglycerol (Membrane lipid)	[M-H]-	null	0,19
Phosphatidylglycerol (Membrane lipid)	[M-H]-	null	0,11
Prostacyclin (Lipid signaling/Prostaglandin)	[M-H]-	null	0,63
Osmolyte (Common in Algae)	[M+H]+	null	0,63
Phenolic acid (Plant hormone/Defense)	[M-H]-	null	0,22
Pyridinecarboxylic acid (Vitamin B6 related)	[M-H]-	null	0,63
Sphingolipid backbone	[M+H]+	null	0,19
Phenylpropanoid pathway	[M+Na]+	null	0,48
Lysine pathway metabolite	[M+H]+	null	0,63
Amino acid derivative (Arginine path)	[M+H]+	null	0,07
Sphingolipid base	[M+Na]+	null	0,15
(Duplicate)	[M+H-H2O]+	null	0,48
Essential Omega-6 Fatty Acid	[M+H]+	null	0,11

Hormone	[M+Na]+	null	0,44
Cyclic amino acid derivative	[M-H]-	null	0,59
(Duplicate entry)	[M-H]-	null	0,33
Trisaccharide (Starch breakdown)	[M+Na]+	null	0,3
Arginine/Putrescine metabolite	[M+Na]+	null	0,63
Biogenic amine	[M-H2O+H]+	null	0,3
Modified nucleoside	[M-H]-	null	0,33
Short-chain organic acid	[M-H]-	null	0,63
Saturated Fatty Acid (14:0)	[M-H]-	null	1
Omega-3 Fatty Acid (18:3)	[M-H]-	null	1
Disaccharide	[M+Na]+	null	0,63
Monounsaturated Fatty Acid (22:1)	[M-H]-	null	0,3
Monounsaturated Fatty Acid (16:1)	[M-H]-	null	0,93
Algal Pigment: (Brown algae/Diatoms)	[M+Na]+	null	0,04
Oxylipin (Oxidized fatty acid)	[M-H]-	null	0,63
Oxylipin	[M+Na]+	null	0,15
Oxylipin (Oxidized lipid)	[M+Na]+	null	0,63
Long-chain Fatty Acid (22:4)	[M-H]-	null	0,48
Saturated Fatty Acid (22:0)	[M-H]-	null	0,15
Saturated Fatty Acid (24:0)	[M-H]-	null	0,52
Saturated Fatty Acid (17:0)	[M-H]-	null	0,3
Monounsaturated Fatty Acid (20:1)	[M-H]-	null	0,48
Monounsaturated Fatty Acid (24:1)	[M-H]-	null	0,44
Monoglyceride (Lipid)	[M+Na]+	null	0,33
Ceramide (Sphingolipid)	[M+CH3COO]-	null	0,3
Ceramide (Sphingolipid)	[M+CH3COO]-	null	0,3
Pyranone derivative	[M+K]+	null	0,07
Sugar ester	[M+Na]+	null	0,3
Glycolipid component	[M+H]+	null	0,41
Sesquiterpene	[M+Na]+	null	0,41
Lipid ester	[M+Na]+	null	0,11
Terpenoid	[M+Na]+	null	0,63
Terpenoid alcohol	[M+Na]+	null	0,48
Sphingolipid isomer	[M+H]+	null	0,15
Oxidized Pelargonic acid (Fatty acid)	[M-H]-	null	0,33
Likely an Amino sugar or Diacid derivative	[M-H]-	null	0,63
Oxidized Stearidonic acid	[M-H2O-H]-	null	0,63
Oxidized short-chain fatty acid	[M-H]-	null	0,63
Oxidized Linoleic acid	[M-H]-	null	0,15
(Duplicate entry)	[M-H]-	null	0,26
Diepoxy or Dihydroxy Fatty acid	[M-H]-	null	0,63
Trihydroxy Fatty Acid (Oxylipin)	[M-H]-	null	0,41
Modified nucleoside	[M+H]+	null	0,15
Methoxyphenol (Antioxidant/Phenolic)	[M-H2O-H]-	null	0,48
(Duplicate entry)	[M-H2O-H]-	null	0,11
Terpenoid derivative	[M+Na]+	null	0,59
Amino acid derivative	[M+Na]+	null	0,52
Diterpenoid ester	[M-H]-	null	0,3
Pro-resolving mediator (Omega-3 derivative)	[M-H]-	null	0,52
Oxylipin (Omega-3 derivative)	[M-H]-	null	0,63

Fatty acid amide (Slip agent in plastics)	[M+H]+	null	0,52
Fatty acid amide (Lipid signaling)	[M+Na]+	null	0,11
Trihydroxy Fatty Acid (Oxylipin)	[M-H]-	null	0,63
Oxylipin (Oxidized fatty acid)	[M+H]+	null	0,07
Lipid amide (Slip agent in plastics)	[M+Na]+	null	0,48
Nucleobase derivative	[M+Na]+	null	0,33
Organic aromatic	[M+Na]+	null	0,63
Brominated phenolic (Algal defense molecule)	[M-H]-	null	0,11

MS/MS assigned	Reference RT	Reference m/z	Formula
TRUE	3,964	87,00877	C3H4O3
TRUE	-1	148,06044	C5H9NO4
TRUE	-1	150,05832	C5H11NO2S
TRUE	-1	205,09715	C11H12N2O2
TRUE	-1	166,08626	C9H11NO2
TRUE	-1	182,08118	C9H11NO3
TRUE	1,797	111,02	C4H4N2O2
TRUE	-1	115,04007	C5H8O3
TRUE	-1	104,10699	C5H14NO
TRUE	-1	132,1019	C6H13NO2
TRUE	0,403	134,04723	C5H5N5
TRUE	0,403	134,04723	C5H5N5
TRUE	-1	116,0706	C5H9NO2
TRUE	-1	133,01425	C4H6O5
TRUE	-1	298,09683	C11H15N5O3S
TRUE	-1	125,03565	C5H6N2O2
TRUE	-1	89,02441	C3H6O3
TRUE	1,88	268,10403	C10H13N5O4
TRUE	29,71	303,23294	C20H32O2
TRUE	29,71	303,23294	C20H32O2
TRUE	3	150,04213	C5H5N5O
TRUE	1,981	124,03931	C6H5NO2
TRUE	2,751	377,14557	C17H20N4O6
TRUE	-1	105,01933	C3H6O4
TRUE	4,732	137,04579	C5H4N4O
TRUE	6,275	267,07349	C10H12N4O5
TRUE	5,22	317,06	C16H12O7
TRUE	7,731	162,11247	C7H15NO3
TRUE	-1	190,04987	C10H7NO3
TRUE	-1	266,08948	C10H13N5O4
TRUE	8,473	883,53418	C47H81O13P
TRUE	-1	151,02615	C5H4N4O2
TRUE	-1	529,15277	C21H30O14
TRUE	0,643	243,06226	C9H12N2O6
TRUE	8,143	743,48688	C40H73O10P
TRUE	8,143	743,48688	C40H73O10P
TRUE	8,359	745,5025	C40H75O10P
TRUE	8,359	745,5025	C40H75O10P
TRUE	-1	351,21771	C20H32O5
TRUE	-1	118,08626	C5H11NO2
TRUE	14,935	137,02441	C7H6O3
TRUE	-1	122,02475	C6H5NO2
TRUE	2,302	302,30536	C18H39NO2
TRUE	6,26	395,11099	C20H20O7
TRUE	-1	162,07608	C6H11NO4
TRUE	-1	146,09241	C5H11N3O2
TRUE	1,533	340,28223	C18H39NO3
TRUE	9,39	303,23001	C18H32O2
TRUE	9,39	303,23001	C18H32O2

TRUE	-1	255,1104	C13H16N2O2
TRUE	1,76	128,035	C5H7NO3
TRUE	1,76	128,035	C5H7NO3
TRUE	-1	527,15826	C18H32O16
TRUE	-1	168,06311	C6H11NO3
TRUE	-1	120,08077	C8H11NO
TRUE	0,902	296,10004	C11H15N5O5
TRUE	-1	103,04007	C4H8O3
TRUE	3,844	227,20166	C14H28O2
TRUE	27,87	277,21732	C18H30O2
TRUE	-1	365,10544	C12H22O11
TRUE	7,621	337,31122	C22H42O2
TRUE	28,89	253,2173	C16H30O2
TRUE	8,23	681,40997	C42H58O6
TRUE	-1	295,22787	C18H32O3
TRUE	-1	317,20871	C18H30O3
TRUE	-1	317,20871	C18H30O3
TRUE	1,053	331,26425	C22H36O2
TRUE	34,37	339,32684	C22H44O2
TRUE	35,22	367,35815	C24H48O2
TRUE	5,329	269,2486	C17H34O2
TRUE	1,062	309,27991	C20H38O2
TRUE	0,95	365,3425	C24H46O2
TRUE	-1	325,23492	C17H34O4
TRUE	12,893	696,61475	C40H79NO4
TRUE	13,072	706,6355	C42H81NO3
TRUE	-1	292,09457	C13H19NO4
TRUE	-1	449,16293	C17H30O12
TRUE	5,4	280,13	C18H17NO2
TRUE	-1	315,15668	C17H24O4
TRUE	-1	351,25058	C19H36O4
TRUE	-1	273,14612	C15H22O3
TRUE	-1	537,30341	C27H46O9
TRUE	7,96	318,29941	C18H39NO3
TRUE	4,9	173,11819	C9H18O3
TRUE	1,85	174,0753	C7H13NO4
TRUE	9,5	291,19449	C18H28O3
TRUE	1,92	133,0497	C5H10O4
TRUE	10,4	293,21222	C18H30O3
TRUE	10,4	293,21222	C18H30O3
TRUE	8,57	311,22159	C18H32O4
TRUE	3,994	327,21771	C18H32O5
TRUE	5,801	298,11459	C11H15N5O5
TRUE	0,615	121,0295	C7H8O3
TRUE	0,615	121,0295	C7H8O3
TRUE	-1	291,15668	C15H24O4
TRUE	3,02	317,10999	C14H18N2O5
TRUE	-1	309,20712	C18H30O4
TRUE	-1	375,21771	C22H32O5
TRUE	-1	317,21222	C20H30O3

TRUE	-1	282,27914	C18H35NO
TRUE	-1	320,25601	C18H35NO2
TRUE	4,5	329,23334	C18H34O5
TRUE	-1	295,22678	C18H30O3
TRUE	-1	415,20911	C22H32O6
TRUE	1,83	193,02	C6H6N2O4
TRUE	4,417	136,07568	C8H9NO
TRUE	7,3	292,84543	C7H4Br2O3

Ontology	INCHIKEY
Alpha-keto acids and derivatives	LCTONWCANYUPML-UHFFFAOYSA-N
Glutamic acid and derivatives	WHUUTDBJXJRKMK-VKHMYHEASA-N
Methionine and derivatives	FFEARJCKVFRZRR-UHFFFAOYSA-N
Indolyl carboxylic acids and derivatives	QIVBCDIJIAJPQS-UHFFFAOYSA-N
Phenylalanine and derivatives	COLNVLDHVKWLRT-UHFFFAOYSA-N
Tyrosine and derivatives	OUYCCCASQSFEME-QMMMGPOBSA-N
Pyrimidones	ISAKRJDGNUQOIC-UHFFFAOYSA-N
Short-chain keto acids and derivatives	KDVFRMMRZOCFLS-UHFFFAOYSA-N
Cholines	OEYIOHPDSNJKLS-UHFFFAOYSA-N
Leucine and derivatives	ROHFNLRQFUQHCH-UHFFFAOYSA-N
6-aminopurines	GFFGJBXGBJISGV-UHFFFAOYSA-N
6-aminopurines	GFFGJBXGBJISGV-UHFFFAOYSA-N
Proline and derivatives	ONIBWKKTOPOVIA-BYPYZUCNSA-N
Beta hydroxy acids and derivatives	BJEPYKJPYRNKOW-REOHCLBHSA-N
5'-deoxy-5'-thionucleosides	WUUGFSXJNOTRMR-IOSLPCCCSA-N
Hydroxypyrimidines	RWQNBRDOKXIBIV-UHFFFAOYSA-N
Alpha hydroxy acids and derivatives	JVTAAEKCZFNVCJ-UHFFFAOYSA-N
Purine nucleosides	OIRDTQYFTABQOQ-UHFFFAOYSA-N
Long-chain fatty acids	YZXBAPSDXZZRGB-DOFZRALJSA-N
Long-chain fatty acids	YZXBAPSDXZZRGB-DOFZRALJSA-N
Purines and purine derivatives	UYTPUPDQBNUYGX-UHFFFAOYSA-N
Pyridinecarboxylic acids	PVNIIMVLHYAWGP-UHFFFAOYSA-N
Flavins	AUNGANRZJHBGPY-SCRDCRAPSA-N
Sugar acids and derivatives	RBNPOMFGQQGHHO-UWTATZPHSA-N
Hypoxanthines	FDGQSTZJBFJUBT-UHFFFAOYSA-N
Purine nucleosides	UGQMRVRMYYASKQ-KQYNXXCUSA-N
6-O-methylated flavonoids	FHHSEFRSDKWJKJ-UHFFFAOYSA-N
Carnitines	PHIQHXFUZVPYII-ZCFIWIBFSA-N
Quinoline carboxylic acids	HCZHHEIFKROPDY-UHFFFAOYSA-N
Purine 2'-deoxyribonucleosides	YKBGVTZYEHREMT-KVQBGUIXSA-N
1-phosphatidyl-1D-myo-inositols	SUYDNPBYMBTSMM-GLWDABGQSA-N
Xanthines	LRFVTYWOQMYALW-UHFFFAOYSA-N
O-glycosyl compounds	XJOKJGJIQOUXDC-UHFFFAOYSA-N
Nucleoside and nucleotide analogues	PTJWIQPHWPFNBW-GBNDHIKLSA-N
Phosphatidylglycerols	PTJBFPPEYDFAAQ-RUGCENDASA-N
Phosphatidylglycerols	PTJBFPPEYDFAAQ-RUGCENDASA-N
Phosphatidylglycerols	ATBOMIWRCZXYSZ-BCTRXSSUSA-N
Phosphatidylglycerols	ATBOMIWRCZXYSZ-BCTRXSSUSA-N
Prostaglandins and related compounds	KAQKFAOMNZTLHT-OZUDYXHBSA-N
Alpha amino acids	KWIUHFFTVRNATP-UHFFFAOYSA-N
Salicylic acids	YGSDEFSMJLZEOE-UHFFFAOYSA-N
Pyridinecarboxylic acids	TWBYWOBDOCUKOW-UHFFFAOYSA-N
1,2-aminoalcohols	OTKJDMGTUTTYMP-ZWKOTPCHSA-N
7-O-methylated flavonoids	LKMNXYDUQXAUCZ-UHFFFAOYSA-N
L-alpha-amino acids	OYIFNHCXNCRBQI-SCSAIBSYSA-N
Gamma amino acids and derivatives	TUHVEAJXIMEOSA-UHFFFAOYSA-N
1,3-aminoalcohols	AERBNCYCJBRYDG-KSZLIROESA-N
Lineolic acids and derivatives	OYHQOLUKZRVURQ-HZJYTTRNSA-N
Lineolic acids and derivatives	OYHQOLUKZRVURQ-HZJYTTRNSA-N

3-alkylindoles	DRLFMBDRBRZALE-UHFFFAOYSA-N
Amino acids	ODHCTXKNWHHXJC-UHFFFAOYNA-N
Amino acids	ODHCTXKNWHHXJC-UHFFFAOYNA-N
Oligosaccharides	FYGDTMLNYKFZSV-PXXRMHSHSA-N
Gamma amino acids and derivatives	UZTFMUBKZQVKLK-UHFFFAOYSA-N
Aralkylamines	ULSIYEODSMZIPX-UHFFFAOYSA-N
Purine nucleosides	OVYNGSFVYRPRCG-KQYNXXCUSA-N
Alpha hydroxy acids and derivatives	BWLBGMIXKSTLSX-UHFFFAOYSA-N
Long-chain fatty acids	TUNFSRHWOTWDNC-UHFFFAOYSA-N
Lineolic acids and derivatives	DTOSIQBPPRVQHS-PDBXOOCHSA-N
O-glycosyl compounds	DLRVVLDZNNYCBX-CQUJWQHSSA-N
Very long-chain fatty acids	DPUOLQHDNGRHBS-KTKRTIGZSA-N
Long-chain fatty acids	SECPZKHBENQXJG-FPLPWBNLSA-N
Xanthophylls	SJWWTRQNNRNTPU-ABBNZJFMSA-N
Lineolic acids and derivatives	NPDSHTNEKLQQIJ-ZJHFMPGASA-N
Lineolic acids and derivatives	JHXAZBBVQSRKJR-QNQPVOBRSA-N
Lineolic acids and derivatives	JHXAZBBVQSRKJR-QNQPVOBRSA-N
Very long-chain fatty acids	TWSWSIQAPQLDBP-DOFZRALJSA-N
Very long-chain fatty acids	UKMSUNONTOPOIO-UHFFFAOYSA-N
Very long-chain fatty acids	QZZGJDVWLFXDLK-UHFFFAOYSA-N
Long-chain fatty acids	KEMQGTRYUADPNZ-UHFFFAOYSA-N
Long-chain fatty acids	BITHHVVYSMSWAG-MDZDMXLPSA-N
Very long-chain fatty acids	GWHCXVQVJPWHRF-KTKRTIGZSA-N
1-monoacylglycerols	DCBSHORRWZKAKO-UHFFFAOYSA-N
Ceramides	FCCZDEAAQZWMGI-NWBJSICCSA-N
Secondary alcohols	FUWLDBVRSLAFFJ-DBOQMASJSA-N
Pyranones and derivatives	LFXMHSJWYXKODM-UHFFFAOYSA-N
Saccharolipids	SFAOCCXTEHOWEV-AGJOLMTFSA-N
Aporphines	JCTYWRARKVGOBK-CQSZACIVSA-N
Sesquiterpenoids	KAMJEJATROMPAT-WUSAYZOBSA-N
Long-chain fatty alcohols	LUIGTZGBXWZJAX-UHFFFAOYSA-N
Sesquiterpenoids	NIQIMYXBAQAIAT-HZMBPMFUSA-N
Glycosylmonoacylglycerols	HUSISCNTLUEZCN-PDBXOOCHSA-N
Lipids	AERBNCYCJBRYDG-UHFFFAOYNA-N
Oxidized fatty acids	XBUXARJOYUQNTC-UHFFFAOYNA-N
Formula predicted	null
Oxidized fatty acids	IYEHRWJNUWAXAD-UHFFFAOYNA-N
Fatty acids	JTEYKUFKXGDTEU-UHFFFAOYNA-N
Long-chain fatty acids	HKSDVVJONLXYKL-UHFFFAOYSA-N
Long-chain fatty acids	HKSDVVJONLXYKL-UHFFFAOYSA-N
Oxidized fatty acids	JGUNZIWGNMQSBM-UHFFFAOYNA-N
Lineolic acids and derivatives	MKYUCBXUUSZMQB-MKZMYESJSA-N
Purine nucleosides	UYARPHAXAJAZLU-KQYNXXCUSA-N
Methoxyphenols	LPYUENQFPVNPHY-UHFFFAOYSA-N
Methoxyphenols	LPYUENQFPVNPHY-UHFFFAOYSA-N
Menthane monoterpenoids	ZIOCYJNRYIRTQD-UHFFFAOYSA-N
Peptides	IAOZJIPTCAWIRG-QWRGUYRKSA-N
Sesquiterpenoids	NLZZWSRYPUGPEO-QUYVSBDASA-N
Very long-chain fatty acids	QBTJOLCUKWLTIC-UZAFJXHNSA-N
Hydroxyeicosapentaenoic acids	WLKCSMCLEKGITB-DBVSHIMFSA-N

Fatty amides	FATBGEAMYMYZAF-MDZDMXLPSA-N
N-acylethanolamines	WFRLANWAASSSFV-FPLPWBNLSA-N
Long-chain fatty acids	DNWUYCUUEGGVPR-CLTKARDFSA-N
Lineolic acids and derivatives	JQXGCBKGIBTCHY-PDBXOOCHSA-N
Diterpenoids	XMWOVKVFIVCVPN-XNTDXEJSSA-N
Alpha amino acids and derivatives	ZFNQFXDDQAEAFI-UHFFFAOYSA-N
Alkyl-phenylketones	CKQHAYFOPRIUOM-UHFFFAOYSA-N
Hydroxybenzoic acid derivatives	PHWAJJWKNLWZGJ-UHFFFAOYSA-N

```
SMILES
CC(=O)C(=O)O
OC(=O)CC[C@H](N)C(O)=O
CSCCC(C(=O)O)N
O=C(O)C(N)CC1=CNC=2C=CC=CC21
O=C(O)C(N)CC=1C=CC=CC1
O=C(O)C(N)CC1=CC=C(O)C=C1
O=C1NC=CC(=O)N1
CCCC(=O)C(O)=O
OCC[N+1](C)(C)C
CC(C)CC(C(=O)O)N
N=1C=NC2=C(N=CNC12)N
N=1C=NC2=C(N=CNC12)N
OC(=O)C([H])(C1)NCC1
C(C(C(=O)O)O)C(=O)O
CSC[C@@H](O1)[C@@H](O)[C@@H](O)[C@@H]1n(c3)c(n2)c(n3)c(N)nc2
CC(=C1)C(=O)NC(=O)N1
CC(O)C(O)=O
OCC3OC(N2C=NC1=C(N=CN=C12)N)C(O)C3(O)
CCCCC/C=C\C/C=C\C/C=C\C/C=C\CCCC(=O)O
CCCCC/C=C\C/C=C\C/C=C\C/C=C\CCCC(=O)O
c1[nH]c2c(n1)nc(nc2O)N
c1cc(cnc1)C(=O)O
Cc1cc2nc3c(nc(=O)[nH]c3=O)n(C[C@H](O)[C@H](O)[C@H](O)CO)c2cc1C
OC[C@@H](O)C(O)=O
OC1=NC=NC=2NC=NC12
OC1=NC=NC2=C1N=CN2C3OC(CO)C(O)C3O
O=C1C=C(OC2=CC(O)=C(OC)C(O)=C12)C=3C=CC(O)=C(O)C3
C[N+](C)(C)C[C@@H](CC(=O)[O-])O
OC(=O)c(c1)nc(c2)c(ccc2)c(O)1
OC[C@@H](O1)[C@@H](O)C[C@@H]1n(c3)c(N=2)c(n3)C(=O)NC(N)2
CCCCCCCCCCCCCCCCCC(=O)OCC(COP(O)(=O)OC1C(O)C(O)C(O)C(O)C1O)OC(=O)CCC\C=C/C\C=C/C\C=C/C\C=C/
C1=NC2=C(N1)C(=O)NC(=O)N2
COC1=CC(=CC(=C1C(=O)OC2C(O)C(O)C(OC2CO)OC3C(O)C(O)C(O)C(O)C3O)C)O
c1c(c(nc(n1)O)O)[C@H]2[C@@H]([C@@H]([C@H](O2)CO)O)O
CCCCCC\C=C/CCCCCCCC(=O)OCC(COP(O)(=O)OCC(O)CO)OC(=O)CCCCCCC\C=C/C\C=C/CCCCC
CCCCCC\C=C/CCCCCCCC(=O)OCC(COP(O)(=O)OCC(O)CO)OC(=O)CCCCCCC\C=C/C\C=C/CCCCC
CCCCCCCCCCCCCCCC(=O)OCC(COP(O)(=O)OCC(O)CO)OC(=O)CCCCCCC\C=C/C\C=C/CCCCC
CCCCCCCCCCCCCCCC(=O)OCC(COP(O)(=O)OCC(O)CO)OC(=O)CCCCCCC\C=C/C\C=C/CCCCC
[H][C@]12C[C@@H](O)[C@H](\C=C\[C@@H](O)CCCCC)[C@@]1([H])C\C(O2)=C\CCCC(O)=O
[O-1]C(=O)C[N+1](C)(C)C
OC(=O)C1=C(O)C=CC=C1
OC(=O)c(c1)ccnc1
CCCCCCCCCCCCCCC[C@H]([C@H](CO)N)O
O=C1C=C(OC=2C=C(OC)C(OC)=C(OC)C12)C=3C=CC(OC)=C(OC)C3
OC(=O)CCCC(N)C(O)=O
C(CC(=O)O)CN=C(N)N
CCCCCCCCCCCCCC[C@H]([C@H]([C@H](CO)N)O)O
O=C(O)CCCCCCCC=CCC=CCCCCC
O=C(O)CCCCCCCC=CCC=CCCCCC
```

COC1=CC2=C(NC=C2CCNC(C)=O)C=C1
O=C(O)C1NC(=O)CC1
O=C(O)C1NC(=O)CC1
OCC1OC(OC2C(O)C(O)C(OC2CO)OC3C(O)C(O)C(O)OC3CO)C(O)C(O)C1O
CC(=O)NCCCC(O)=O
NCC(O)C1=CC=CC=C1
CO[C@@H]1[C@@H]([C@H](O[C@H]1n2cnc3c2nc([nH]c3=O)N)CO)O
OC(=O)C(C)(C)O
O=C(O)CCCCCCCCCCCCC
OC(=O)CCCCCCC/C=C\C/C=C\C/C=C\CC
OCC1OC(OCC2OC(O)C(O)C(O)C2O)C(O)C(O)C1O
CCCCCCCC/C=C\CCCCCCCCCCCC(=O)O
CCCCCC/C=C\CCCCCCCC(=O)O
O=C(OC1CC(O)(C(=C=CC(=CC=CC(=CC=CC=C(C=CC=C(C(=O)CC23OC3(C)CC(O)CC2(C)C)C)C)C)C)C(C)(C)C1)C)C
CCCCC\C=C/C=C/C(O)CCCCCCCC(O)=O
CCCCCC(=O)/C=C\C=C\CCCCCCCC(=O)O
CCCCCC(=O)/C=C\C=C\CCCCCCCC(=O)O
O=C(O)CCCCCC=CCC=CCC=CCC=CCCCCC
CCCCCCCCCCCCCCCCCCCCCC(=O)O
CCCCCCCCCCCCCCCCCCCCCCCC(=O)O
CCCCCCCCCCCCCCCCC(=O)O
O=C(O)CCCCCCCCCC=CCCCCCCCC
O=C(O)CCCCCCCCCCCCCC=CCCCCCCCC
CCCCCCCCCCCCCC(=O)OCC(CO)O
CCCCCCCCCCCCCCCCCCCCC(O)C(=O)NC(CO)C(O)\C=C\CCCCCCCCCCCCC
CCCCCCCCCCCCC\C=C\C(O)C(CO)NC(=O)CCCCCCCCC\C=C/CCCCCCCCCCCCC
CCC(C)C(NC(C)=O)C1=CC(OC)=CC(=O)O1
CCC(C)C(=O)O[C@H]1[C@H](O)[C@@H](CO)O[C@H](O[C@H]2O[C@H](CO)[C@@H](O)[C@H](O)[C@H]2O)[C[illegible]
O1C=2C=C3C4=C(C2OC1)C5=CC=CC=C5CC4N(C)CC3
CC(=O)OC[C@]1(C)C[C@H]2[C@H]1CC(=O)\C(=C/CCC2=C)CO
CC(=O)OC(CO)CC(O)CCCCCCCCCCCCC=C
C[C@H]\1CC[C@@H](C\C=C1CCC(C)=O)C(=C)C(O)=O
CC\C=C/C/C=C\C\C=C/CCCCCCCC(=O)OCC(O)COC1OC(CO)C(O)C(O)C1O
OCC(N)C(O)C(O)CCCCCCCCCCCCCC
O=C(O)CC(O)CCCCCC
null
O=C(O)CCCCCCCC1C(=O)C=CC1(CC=CCC)
O=C(O)C(O)C(O)(C)C
O=C(O)CCCCCCCC=CCC=CCC1OC1(CC)
O=C(O)CCCCCCCC=CCC=CCC1OC1(CC)
O=C(O)CCCCCCCC(OO)C=CC=CCCCCC
O=C(O)CCCCCCCC(O)C=CC(O)C(O)CC=CCC
N=C1N=C(O)C=2N=CN(C2N1)C3OC(CO)C(OC)C3O
OC1=CC=CC(OC)=C1O
OC1=CC=CC(OC)=C1O
O=C(O)CC(C)C1(C(=O)C(C)C)CC(=O)C(C)CC1
O=C(O)CC(N)C(=O)NC(C(=O)OC)CC=1C=CC=CC1
O=C(OC)C=CC(=CCC(O)C(=CCCC(C)C(O)C)C)C
C(=C/[C@@H](C\C=C/C=C/[C@H](C/C=C\CC)O)O)\C=C\C=C/[C@H](CCC(O)=O)O
CCC=CCC(O)C=CC=CCC=CCC=CCCCC(O)=O

CCCCCCCCC=CCCCCCCCC(=O)N

CCCCCC/C=C\CCCCCCCC(=O)NCCO

O=C(O)CCCCCCCC(O)CCC(O)C(O)CC=CCC

O=C(O)C(O)CCCCCCC=CCC=CCC=CCC

CC(=O)OC\\C(CCC1C(C=O)=CC(O)C2C(C)(C)CCCC12C)=C\\C(O)=O

O=C1C=CN(C(=O)N1)CC(=O)O

CC(=O)C1=CC(N)=CC=C1

O=C(O)c1cc(c(O)c(Br)c1)Br

Annotation tag (VS1.0)	RT matched	m/z matched	MS/MS matched
530	FALSE	TRUE	FALSE
430	FALSE	TRUE	TRUE
430	FALSE	TRUE	TRUE
430	FALSE	TRUE	TRUE
430	FALSE	TRUE	TRUE
430	FALSE	TRUE	TRUE
530	FALSE	TRUE	FALSE
530	FALSE	TRUE	FALSE
430	FALSE	TRUE	TRUE
530	FALSE	TRUE	FALSE
530	FALSE	TRUE	FALSE
530	FALSE	TRUE	FALSE
530	FALSE	TRUE	FALSE
530	FALSE	TRUE	FALSE
530	FALSE	TRUE	FALSE
530	FALSE	TRUE	FALSE
530	FALSE	TRUE	FALSE
430	FALSE	TRUE	TRUE
430	FALSE	TRUE	TRUE
530	FALSE	TRUE	FALSE
530	FALSE	TRUE	FALSE
530	FALSE	TRUE	FALSE
530	FALSE	TRUE	FALSE
530	FALSE	TRUE	FALSE
430	FALSE	TRUE	TRUE
530	FALSE	TRUE	FALSE
530	FALSE	TRUE	FALSE
530	FALSE	TRUE	FALSE
430	FALSE	TRUE	TRUE
530	FALSE	TRUE	FALSE
530	FALSE	TRUE	FALSE
530	FALSE	TRUE	FALSE
530	FALSE	TRUE	FALSE
530	FALSE	TRUE	FALSE
530	FALSE	TRUE	FALSE
530	FALSE	TRUE	FALSE
430	FALSE	TRUE	TRUE
430	FALSE	TRUE	TRUE
530	FALSE	TRUE	FALSE
430	FALSE	TRUE	TRUE
530	FALSE	TRUE	FALSE
530	FALSE	TRUE	FALSE
530	FALSE	TRUE	FALSE
530	FALSE	TRUE	FALSE
430	FALSE	TRUE	TRUE
530	FALSE	TRUE	FALSE
530	FALSE	TRUE	FALSE
530	FALSE	TRUE	FALSE
530	FALSE	TRUE	FALSE

530	FALSE	TRUE	FALSE
530	FALSE	TRUE	FALSE
530	FALSE	TRUE	FALSE
530	FALSE	TRUE	FALSE
530	FALSE	TRUE	FALSE
530	FALSE	TRUE	FALSE
530	FALSE	TRUE	FALSE
530	FALSE	TRUE	FALSE
530	FALSE	TRUE	FALSE
530	FALSE	TRUE	FALSE
530	FALSE	TRUE	FALSE
530	FALSE	TRUE	FALSE
530	FALSE	TRUE	FALSE
530	FALSE	TRUE	FALSE
530	FALSE	TRUE	FALSE
530	FALSE	TRUE	FALSE
430	FALSE	TRUE	TRUE
530	FALSE	TRUE	FALSE
530	FALSE	TRUE	FALSE
530	FALSE	TRUE	FALSE
530	FALSE	TRUE	FALSE
530	FALSE	TRUE	FALSE
530	FALSE	TRUE	FALSE
530	FALSE	TRUE	FALSE
530	FALSE	TRUE	FALSE
530	FALSE	TRUE	FALSE
530	FALSE	TRUE	FALSE
530	FALSE	TRUE	FALSE
530	FALSE	TRUE	FALSE
530	FALSE	TRUE	FALSE
530	FALSE	TRUE	FALSE
530	FALSE	TRUE	FALSE
430	FALSE	TRUE	TRUE
530	FALSE	TRUE	FALSE
530	FALSE	TRUE	FALSE
530	FALSE	TRUE	FALSE
530	FALSE	TRUE	FALSE
530	FALSE	TRUE	FALSE
530	FALSE	TRUE	FALSE
530	FALSE	TRUE	FALSE
530	FALSE	TRUE	FALSE
530	FALSE	TRUE	FALSE
530	FALSE	TRUE	FALSE
530	FALSE	TRUE	FALSE
530	FALSE	TRUE	FALSE
530	FALSE	TRUE	FALSE
530	FALSE	TRUE	FALSE
530	FALSE	TRUE	FALSE
530	FALSE	TRUE	FALSE
530	FALSE	TRUE	FALSE

430	FALSE	TRUE	TRUE
530	FALSE	TRUE	FALSE
530	FALSE	TRUE	FALSE
530	FALSE	TRUE	FALSE
530	FALSE	TRUE	FALSE
530	FALSE	TRUE	FALSE
530	FALSE	TRUE	FALSE
530	FALSE	TRUE	FALSE

Comment
Normalized unit: NormalizedByInternalStandardPeakHeight; Annotation method: MSMS-Public_all-neg-VS19_
Normalized unit: NormalizedByInternalStandardPeakHeight; Annotation method: MSMS-Public_all-pos-VS19_
Normalized unit: NormalizedByInternalStandardPeakHeight; Annotation method: MSMS-Public_all-pos-VS19_
Normalized unit: NormalizedByInternalStandardPeakHeight; Annotation method: MSMS-Public_all-pos-VS19_
Normalized unit: NormalizedByInternalStandardPeakHeight; Annotation method: MSMS-Public_all-pos-VS19_
Normalized unit: NormalizedByInternalStandardPeakHeight; Annotation method: MSMS-Public_all-pos-VS19_
Normalized unit: NormalizedByInternalStandardPeakHeight; Annotation method: MSMS-Public_all-neg-VS19_
Normalized unit: NormalizedByInternalStandardPeakHeight; Annotation method: MSMS-Public_all-neg-VS19_
Normalized unit: NormalizedByInternalStandardPeakHeight; Annotation method: MSMS-Public_all-pos-VS19_
Normalized unit: NormalizedByInternalStandardPeakHeight; Annotation method: MSMS-Public_all-pos-VS19_
Normalized unit: NormalizedByInternalStandardPeakHeight; Annotation method: MSMS-Public_all-neg-VS19_
Normalized unit: NormalizedByInternalStandardPeakHeight; Annotation method: MSMS-Public_all-neg-VS19_
Normalized unit: NormalizedByInternalStandardPeakHeight; Annotation method: MSMS-Public_all-pos-VS19_
Normalized unit: NormalizedByInternalStandardPeakHeight; Annotation method: MSMS-Public_all-neg-VS19_
Normalized unit: NormalizedByInternalStandardPeakHeight; Annotation method: MSMS-Public_all-pos-VS19_
Normalized unit: NormalizedByInternalStandardPeakHeight; Annotation method: MSMS-Public_all-neg-VS19_
Normalized unit: NormalizedByInternalStandardPeakHeight; Annotation method: MSMS-Public_all-neg-VS19_
Normalized unit: NormalizedByInternalStandardPeakHeight; Annotation method: MSMS-Public_all-pos-VS19_
Normalized unit: NormalizedByInternalStandardPeakHeight; Annotation method: MSMS-Public_all-neg-VS19_
Normalized unit: NormalizedByInternalStandardPeakHeight; Annotation method: MSMS-Public_all-neg-VS19_
Normalized unit: NormalizedByInternalStandardPeakHeight; Annotation method: MSMS-Public_all-neg-VS19_
Normalized unit: NormalizedByInternalStandardPeakHeight; Annotation method: MSMS-Public_all-pos-VS19_
Normalized unit: NormalizedByInternalStandardPeakHeight; Annotation method: MSMS-Public_all-pos-VS19_
Normalized unit: NormalizedByInternalStandardPeakHeight; Annotation method: MSMS-Public_all-neg-VS19_
Normalized unit: NormalizedByInternalStandardPeakHeight; Annotation method: MSMS-Public_all-pos-VS19_
Normalized unit: NormalizedByInternalStandardPeakHeight; Annotation method: MSMS-Public_all-neg-VS19_
Normalized unit: NormalizedByInternalStandardPeakHeight; Annotation method: MSMS-Public_all-pos-VS19_
Normalized unit: NormalizedByInternalStandardPeakHeight; Annotation method: MSMS-Public_all-pos-VS19_
Normalized unit: NormalizedByInternalStandardPeakHeight; Annotation method: MSMS-Public_all-pos-VS19_
Normalized unit: NormalizedByInternalStandardPeakHeight; Annotation method: MSMS-Public_all-neg-VS19_
Normalized unit: NormalizedByInternalStandardPeakHeight; Annotation method: MSMS-Public_all-neg-VS19_
Normalized unit: NormalizedByInternalStandardPeakHeight; Annotation method: MSMS-Public_all-neg-VS19_
Normalized unit: NormalizedByInternalStandardPeakHeight; Annotation method: MSMS-Public_all-pos-VS19_
Normalized unit: NormalizedByInternalStandardPeakHeight; Annotation method: MSMS-Public_all-neg-VS19_
Normalized unit: NormalizedByInternalStandardPeakHeight; Annotation method: MSMS-Public_all-neg-VS19_
Normalized unit: NormalizedByInternalStandardPeakHeight; Annotation method: MSMS-Public_all-neg-VS19_
Normalized unit: NormalizedByInternalStandardPeakHeight; Annotation method: MSMS-Public_all-neg-VS19_
Normalized unit: NormalizedByInternalStandardPeakHeight; Annotation method: MSMS-Public_all-neg-VS19_
Normalized unit: NormalizedByInternalStandardPeakHeight; Annotation method: MSMS-Public_all-neg-VS19_
Normalized unit: NormalizedByInternalStandardPeakHeight; Annotation method: MSMS-Public_all-pos-VS19_
Normalized unit: NormalizedByInternalStandardPeakHeight; Annotation method: MSMS-Public_all-neg-VS19_
Normalized unit: NormalizedByInternalStandardPeakHeight; Annotation method: MSMS-Public_all-neg-VS19_
Normalized unit: NormalizedByInternalStandardPeakHeight; Annotation method: MSMS-Public_all-pos-VS19_
Normalized unit: NormalizedByInternalStandardPeakHeight; Annotation method: MSMS-Public_all-pos-VS19_
Normalized unit: NormalizedByInternalStandardPeakHeight; Annotation method: MSMS-Public_all-pos-VS19_
Normalized unit: NormalizedByInternalStandardPeakHeight; Annotation method: MSMS-Public_all-pos-VS19_
Normalized unit: NormalizedByInternalStandardPeakHeight; Annotation method: MSMS-Public_all-pos-VS19_
Normalized unit: NormalizedByInternalStandardPeakHeight; Annotation method: MSMS-Public_all-pos-VS19_
Normalized unit: NormalizedByInternalStandardPeakHeight; Annotation method: MSMS-Public_all-pos-VS19_

Normalized unit: NormalizedByInternalStandardPeakHeight; Annotation method: MSMS-Public_all-pos-VS19_
Normalized unit: NormalizedByInternalStandardPeakHeight; Annotation method: MSMS-Public_all-neg-VS19_
Normalized unit: NormalizedByInternalStandardPeakHeight; Annotation method: MSMS-Public_all-neg-VS19_
Normalized unit: NormalizedByInternalStandardPeakHeight; Annotation method: MSMS-Public_all-pos-VS19_
Normalized unit: NormalizedByInternalStandardPeakHeight; Annotation method: MSMS-Public_all-pos-VS19_
Normalized unit: NormalizedByInternalStandardPeakHeight; Annotation method: MSMS-Public_all-pos-VS19_
Normalized unit: NormalizedByInternalStandardPeakHeight; Annotation method: MSMS-Public_all-neg-VS19_
Normalized unit: NormalizedByInternalStandardPeakHeight; Annotation method: MSMS-Public_all-neg-VS19_
Normalized unit: NormalizedByInternalStandardPeakHeight; Annotation method: MSMS-Public_all-neg-VS19_
Normalized unit: NormalizedByInternalStandardPeakHeight; Annotation method: MSMS-Public_all-neg-VS19_
Normalized unit: NormalizedByInternalStandardPeakHeight; Annotation method: MSMS-Public_all-pos-VS19_
Normalized unit: NormalizedByInternalStandardPeakHeight; Annotation method: MSMS-Public_all-neg-VS19_
Normalized unit: NormalizedByInternalStandardPeakHeight; Annotation method: MSMS-Public_all-neg-VS19_
Normalized unit: NormalizedByInternalStandardPeakHeight; Annotation method: MSMS-Public_all-pos-VS19_
Normalized unit: NormalizedByInternalStandardPeakHeight; Annotation method: MSMS-Public_all-neg-VS19_
Normalized unit: NormalizedByInternalStandardPeakHeight; Annotation method: MSMS-Public_all-pos-VS19_
Normalized unit: NormalizedByInternalStandardPeakHeight; Annotation method: MSMS-Public_all-pos-VS19_
Normalized unit: NormalizedByInternalStandardPeakHeight; Annotation method: MSMS-Public_all-neg-VS19_
Normalized unit: NormalizedByInternalStandardPeakHeight; Annotation method: MSMS-Public_all-neg-VS19_
Normalized unit: NormalizedByInternalStandardPeakHeight; Annotation method: MSMS-Public_all-neg-VS19_
Normalized unit: NormalizedByInternalStandardPeakHeight; Annotation method: MSMS-Public_all-neg-VS19_
Normalized unit: NormalizedByInternalStandardPeakHeight; Annotation method: MSMS-Public_all-neg-VS19_
Normalized unit: NormalizedByInternalStandardPeakHeight; Annotation method: MSMS-Public_all-neg-VS19_
Normalized unit: NormalizedByInternalStandardPeakHeight; Annotation method: MSMS-Public_all-pos-VS19_
Normalized unit: NormalizedByInternalStandardPeakHeight; Annotation method: MSMS-Public_all-neg-VS19_
Normalized unit: NormalizedByInternalStandardPeakHeight; Annotation method: MSMS-Public_all-neg-VS19_
Normalized unit: NormalizedByInternalStandardPeakHeight; Annotation method: MSMS-Public_all-pos-VS19_
Normalized unit: NormalizedByInternalStandardPeakHeight; Annotation method: MSMS-Public_all-pos-VS19_
Normalized unit: NormalizedByInternalStandardPeakHeight; Annotation method: MSMS-Public_all-pos-VS19_
Normalized unit: NormalizedByInternalStandardPeakHeight; Annotation method: MSMS-Public_all-pos-VS19_
Normalized unit: NormalizedByInternalStandardPeakHeight; Annotation method: MSMS-Public_all-pos-VS19_
Normalized unit: NormalizedByInternalStandardPeakHeight; Annotation method: MSMS-Public_all-pos-VS19_
Normalized unit: NormalizedByInternalStandardPeakHeight; Annotation method: MSMS-Public_all-pos-VS19_
Normalized unit: NormalizedByInternalStandardPeakHeight; Annotation method: MSMS-Public_all-pos-VS19_
Normalized unit: NormalizedByInternalStandardPeakHeight; Annotation method: MSMS-Public_all-neg-VS19_
Normalized unit: NormalizedByInternalStandardPeakHeight; Annotation method: MSMS-Public_all-neg-VS19_
Normalized unit: NormalizedByInternalStandardPeakHeight; Annotation method: MSMS-Public_all-neg-VS19_
Normalized unit: NormalizedByInternalStandardPeakHeight; Annotation method: MSMS-Public_all-neg-VS19_
Normalized unit: NormalizedByInternalStandardPeakHeight; Annotation method: MSMS-Public_all-neg-VS19_
Normalized unit: NormalizedByInternalStandardPeakHeight; Annotation method: MSMS-Public_all-neg-VS19_
Normalized unit: NormalizedByInternalStandardPeakHeight; Annotation method: MSMS-Public_all-neg-VS19_
Normalized unit: NormalizedByInternalStandardPeakHeight; Annotation method: MSMS-Public_all-neg-VS19_
Normalized unit: NormalizedByInternalStandardPeakHeight; Annotation method: MSMS-Public_all-pos-VS19_
Normalized unit: NormalizedByInternalStandardPeakHeight; Annotation method: MSMS-Public_all-neg-VS19_
Normalized unit: NormalizedByInternalStandardPeakHeight; Annotation method: MSMS-Public_all-neg-VS19_
Normalized unit: NormalizedByInternalStandardPeakHeight; Annotation method: MSMS-Public_all-pos-VS19_
Normalized unit: NormalizedByInternalStandardPeakHeight; Annotation method: MSMS-Public_all-pos-VS19_
Normalized unit: NormalizedByInternalStandardPeakHeight; Annotation method: MSMS-Public_all-neg-VS19_
Normalized unit: NormalizedByInternalStandardPeakHeight; Annotation method: MSMS-Public_all-neg-VS19_
Normalized unit: NormalizedByInternalStandardPeakHeight; Annotation method: MSMS-Public_all-neg-VS19_

Normalized unit: NormalizedByInternalStandardPeakHeight; Annotation method: MSMS-Public_all-pos-VS19_
Normalized unit: NormalizedByInternalStandardPeakHeight; Annotation method: MSMS-Public_all-pos-VS19_
Normalized unit: NormalizedByInternalStandardPeakHeight; Annotation method: MSMS-Public_all-neg-VS19_
Normalized unit: NormalizedByInternalStandardPeakHeight; Annotation method: MSMS-Public_all-pos-VS19_
Normalized unit: NormalizedByInternalStandardPeakHeight; Annotation method: MSMS-Public_all-pos-VS19_
Normalized unit: NormalizedByInternalStandardPeakHeight; Annotation method: MSMS-Public_all-pos-VS19_
Normalized unit: NormalizedByInternalStandardPeakHeight; Annotation method: MSMS-Public_all-pos-VS19_
Normalized unit: NormalizedByInternalStandardPeakHeight; Annotation method: MSMS-Public_all-neg-VS19_

Manually modified for quantification	Manually modified for annotation
FALSE	FALSE
FALSE	FALSE
FALSE	FALSE
FALSE	FALSE
FALSE	FALSE
FALSE	FALSE
FALSE	FALSE
FALSE	FALSE
FALSE	FALSE
FALSE	FALSE
FALSE	FALSE
FALSE	FALSE
FALSE	FALSE
FALSE	FALSE
FALSE	FALSE
FALSE	FALSE
FALSE	FALSE
FALSE	FALSE
FALSE	FALSE
FALSE	FALSE
FALSE	FALSE
FALSE	FALSE
FALSE	FALSE
FALSE	FALSE
FALSE	FALSE
FALSE	FALSE
FALSE	FALSE
FALSE	FALSE
FALSE	FALSE
FALSE	FALSE
FALSE	FALSE
FALSE	FALSE
FALSE	FALSE
FALSE	FALSE
FALSE	FALSE
FALSE	FALSE
FALSE	FALSE
FALSE	FALSE
FALSE	FALSE
FALSE	FALSE
FALSE	FALSE
FALSE	FALSE
FALSE	FALSE
FALSE	FALSE
FALSE	FALSE
FALSE	FALSE
FALSE	FALSE
FALSE	FALSE
FALSE	FALSE

FALSE	FALSE
FALSE	FALSE
FALSE	FALSE
FALSE	FALSE
FALSE	FALSE
FALSE	FALSE
FALSE	FALSE
FALSE	FALSE
FALSE	FALSE
FALSE	FALSE
FALSE	FALSE
FALSE	FALSE
FALSE	FALSE
FALSE	FALSE
FALSE	FALSE
FALSE	FALSE
FALSE	FALSE
FALSE	FALSE
FALSE	FALSE
FALSE	FALSE
FALSE	FALSE
FALSE	FALSE
FALSE	FALSE
FALSE	FALSE
FALSE	FALSE
FALSE	FALSE
FALSE	FALSE
FALSE	FALSE
FALSE	FALSE
FALSE	FALSE
FALSE	FALSE
FALSE	FALSE
FALSE	FALSE
FALSE	FALSE
FALSE	FALSE
FALSE	FALSE
FALSE	FALSE
FALSE	FALSE
FALSE	FALSE
FALSE	FALSE
FALSE	FALSE
FALSE	FALSE
FALSE	FALSE
FALSE	FALSE
FALSE	FALSE
FALSE	FALSE
FALSE	FALSE
FALSE	FALSE
FALSE	FALSE
FALSE	FALSE

FALSE	FALSE
FALSE	FALSE
FALSE	FALSE
FALSE	FALSE
FALSE	FALSE
FALSE	FALSE
FALSE	FALSE
FALSE	FALSE

Isotope tracking parent ID	Isotope tracking weight number	RT similarity	m/z similarity
3	0	0	0,98
63	0	0	0,99
67	0	0	0,95
193	0	0	0,98
96	0	0	0,98
133	0	0	0,98
13	0	0	0,98
15	0	0	0,96
12	0	0	0,99
37	0	0	1
43	0	0	0,97
44	0	0	0,98
27	0	0	0,98
41	0	0	0,98
499	0	0	0,95
24	0	0	0,98
4	0	0	0,98
402	0	0	0,98
490	0	0	0,97
491	0	0	0,96
78	0	0	0,97
31	0	0	1
882	0	0	1
11	0	0	0,97
47	0	0	0,98
354	0	0	0,98
560	0	0	0,8
88	0	0	0,98
156	0	0	1
351	0	0	0,98
1845	0	0	0,98
80	0	0	0,97
1467	0	0	0,95
277	0	0	0,97
1702	0	0	0,92
1703	0	0	0,88
1710	0	0	0,98
1711	0	0	0,97
628	0	0	0,96
29	0	0	1
48	0	0	0,97
23	0	0	0,97
511	0	0	0,99
959	0	0	0,7
87	0	0	0,99
58	0	0	0,96
655	0	0	0,98
516	0	0	1
517	0	0	1

367	0	0	0,74
32	0	0	0,98
33	0	0	0,98
1460	0	0	1
98	0	0	0,62
30	0	0	0,97
464	0	0	0,96
10	0	0	0,98
229	0	0	0,98
383	0	0	0,97
767	0	0	0,98
595	0	0	0,98
307	0	0	0,98
1891	0	0	1
458	0	0	0,98
565	0	0	1
564	0	0	0,98
575	0	0	0,97
599	0	0	0,96
669	0	0	0,94
363	0	0	0,97
518	0	0	0,93
667	0	0	0,98
600	0	0	0,95
1623	0	0	1
1635	0	0	0,97
485	0	0	1
1180	0	0	0,91
444	0	0	1
553	0	0	0,93
701	0	0	0,65
422	0	0	0,98
1505	0	0	1
569	0	0	0,99
106	0	0	0,98
107	0	0	1
445	0	0	1
42	0	0	1
453	0	0	0,97
454	0	0	0,97
521	0	0	1
564	0	0	0,99
500	0	0	0,93
21	0	0	0,98
22	0	0	0,98
482	0	0	0,99
561	0	0	0,98
515	0	0	0,98
689	0	0	0,77
533	0	0	0,95

449	0	0	0,97
581	0	0	0,96
570	0	0	1
493	0	0	0,99
1043	0	0	0,99
166	0	0	0,62
44	0	0	0,78
448	0	0	0,97

Simple dot product	Weighted dot product	Reverse dot product	Matched peaks count
0,931	0,938	0,904	1
0,972	0,949	0,958	4
0,835	0,839	0,94	6
0,857	0,909	0,948	11
0,945	0,888	0,924	6
0,938	0,938	0,977	10
0,996	0,938	0,933	2
0,114	0,136	0,866	1
0,969	0,953	0,966	3
0,935	0,88	0,938	2
0,42	0,571	0,881	1
0,926	0,938	0,881	1
1	0,938	0,938	2
0,158	0,204	0,874	1
0,958	0,934	0,904	2
0,72	0,75	0,866	1
1	0,866	0,866	1
0,958	0,904	0,924	4
0,999	0,938	0,938	3
0,489	0,516	0,845	1
0,971	0,937	0,918	2
1	0,866	0,866	1
0,581	0,548	0,855	1
0,181	0,198	0,873	1
0,946	0,945	0,971	4
1	0,938	0,938	2
0,081	0,084	0,866	1
0,568	0,558	0,866	2
0,843	0,875	0,865	3
0,82	0,938	0,803	1
0,601	0,85	0,886	1
0,999	0,937	0,937	2
0,374	0,398	0,925	2
0,903	0,938	0,876	1
0,711	0,867	0,846	2
0,782	0,935	0,86	2
0,862	0,895	0,907	3
0,91	0,93	0,903	3
0,967	0,866	0,844	1
0,971	0,944	0,951	3
0,929	0,938	0,845	1
0,225	0,244	0,936	2
0,775	0,9	0,888	2
0,34	0,327	0,866	1
0,841	0,889	0,974	5
0,409	0,593	0,834	2
0,753	0,676	0,866	1
0,789	0,753	0,866	1
0,693	0,604	0,866	1

0,275	0,294	0,866	1
0,568	0,618	0,866	1
0,379	0,426	0,866	1
0,883	0,929	0,84	2
0,945	0,823	0,863	1
0,907	0,904	0,861	2
1	0,866	0,866	1
0,702	0,617	0,866	1
0,964	0,836	0,866	1
1	0,938	0,938	2
0,992	0,938	0,93	2
0,979	0,866	0,851	1
1	0,938	0,938	2
0,169	0,151	0,866	1
0,784	0,884	0,877	1
0,724	0,714	0,93	2
0,964	0,913	0,937	3
0,953	0,866	0,842	1
0,731	0,679	0,866	1
0,979	0,922	0,938	2
1	0,866	0,866	1
0,848	0,913	0,863	2
1	0,866	0,866	1
0,303	0,301	0,843	1
0,803	0,938	0,814	1
0,513	0,635	0,804	2
0,677	0,66	0,804	1
0,384	0,461	0,868	1
0,799	0,733	0,855	1
0,681	0,619	0,833	1
0,572	0,582	0,899	1
0,872	0,808	0,836	2
0,753	0,744	0,928	3
0,705	0,78	0,829	1
0,949	0,866	0,833	1
0,875	0,938	0,847	1
0,164	0,202	0,857	1
0,441	0,634	0,832	1
0,67	0,685	0,9	1
0,817	0,823	0,9	1
0,703	0,82	0,868	1
0,508	0,608	0,849	1
0,904	0,797	0,866	1
0,887	0,923	0,825	1
0,877	0,904	0,825	1
0,919	0,912	0,91	2
0,348	0,351	0,932	2
0,738	0,86	0,876	2
0,822	0,885	0,906	1
0,843	0,793	0,845	1

0,922	0,94	0,945	22
0,729	0,712	0,933	2
0,982	0,866	0,855	1
0,241	0,206	0,866	1
0,511	0,562	0,843	1
0,108	0,116	0,866	1
0,588	0,565	0,866	1
0,306	0,366	0,86	1

Matched peaks percentage	Total score	S/N average	Spectrum reference file name
0,25	1,766	1685,36	NEG_H2O_3
1	1,944	179,03	POS_QC_4
0,86	1,771	1834,96	POS_H2O_2
0,73	1,812	649,95	POS_H2O_1
1	1,88	6962,22	POS_H2O_2
1	1,921	5444,49	POS_H2O_4
0,67	1,877	16363,99	NEG_MeOH-1
1	1,059	1399,77	NEG_H2O_1
1	1,943	5350,33	POS_EtOH-1
1	1,885	3945,72	POS_MeOH-4
0,5	1,357	1608,46	NEG_MeOH-3
0,5	1,8	2685,83	NEG_QC_2
1	1,94	1530,41	POS_EtOH-2
0,33	1,017	1841,91	NEG_H2O_2
0,67	1,833	1561,08	POS_EtOH-4
1	1,68	3549,12	NEG_H2O_1
1	1,878	5971,98	NEG_MeOH-1
1	1,897	21064,14	POS_QC_3
1	1,932	75979,47	NEG_EtOH-4
0,33	1,322	2414,19	NEG_QC_3
0,5	1,828	3587,08	NEG_H2O_2
1	1,887	2217,58	POS_EtOH-4
0,5	1,432	1503,53	POS_H2O_2
0,33	1,021	1292,92	NEG_QC_3
1	1,928	9470,15	POS_MeOH-4
1	1,937	7257,13	NEG_QC_4
1	0,94	3753,99	POS_QC_3
1	1,507	1937,73	POS_EtOH-4
0,43	1,719	94,07	POS_QC_3
0,2	1,672	3585,32	NEG_H2O_2
0,08	1,525	2469,33	NEG_MeOH-2
1	1,932	81,4	NEG_H2O_3
0,4	1,237	1111,38	POS_EtOH-4
0,33	1,755	16,69	NEG_MeOH-1
0,15	1,558	3065,46	NEG_EtOH-4
0,15	1,614	2751,13	NEG_EtOH-1
0,38	1,733	5708,96	NEG_EtOH-2
0,38	1,768	5612,9	NEG_EtOH-1
0,2	1,711	2198,36	NEG_H2O_3
1	1,939	13100,28	POS_H2O_2
0,5	1,782	1204,03	NEG_H2O_1
1	1,2	2962,84	NEG_H2O_2
0,14	1,65	1081,36	POS_EtOH-2
1	1,139	1876,57	POS_MeOH-4
1	1,85	7761,53	POS_QC_1
0,25	1,301	1156,47	POS_QC_1
1	1,659	1530,17	POS_EtOH-1
1	1,726	3170,51	POS_EtOH-3
1	1,604	1203,02	POS_EtOH-1

1	1,109	13,55	POS_QC_2
1	1,538	16,77	NEG_MeOH-1
1	1,35	21,72	NEG_EtOH-1
0,4	1,752	2621,7	POS_H2O_2
0,5	1,565	53111,97	POS_QC_2
0,25	1,721	2537,62	POS_QC_5
1	1,868	2067,54	NEG_H2O_2
1	1,605	4824,97	NEG_MeOH-4
1	1,844	13490,01	NEG_EtOH-1
1	1,933	77710,65	NEG_EtOH-3
0,67	1,875	5223,41	POS_H2O_4
0,33	1,752	2925,32	NEG_MeOH-3
1	1,938	26227,51	NEG_EtOH-1
1	1,114	1021,69	POS_EtOH-1
0,25	1,659	6954,12	NEG_QC_1
0,67	1,638	1524,48	POS_QC_1
1	1,906	3341,92	POS_MeOH-2
0,2	1,711	9275,12	NEG_EtOH-1
1	1,642	1690,58	NEG_EtOH-2
1	1,9	5065,11	NEG_EtOH-1
1	1,874	2403,35	NEG_EtOH-1
0,29	1,681	4626,58	NEG_MeOH-2
1	1,876	7987,95	NEG_EtOH-4
0,33	1,114	1788,86	POS_MeOH-2
0,06	1,65	2541,63	NEG_EtOH-3
0,12	1,346	3505,48	NEG_EtOH-1
0,25	1,476	1187,81	POS_H2O_3
0,2	1,2	1384,54	POS_MeOH-2
0,5	1,633	3473,28	POS_H2O_2
0,25	1,436	1340,9	POS_EtOH-1
0,5	1,282	1562,13	POS_H2O_1
0,2	1,642	5580,15	POS_QC_2
1	1,725	2447,62	POS_MeOH-3
0,5	1,6	1562,58	POS_EtOH-2
0,33	1,733	2407,2	NEG_H2O_1
0,5	1,594	2668,21	NEG_H2O_2
0,5	1,052	2089,42	NEG_QC_1
0,25	1,355	2060,96	NEG_H2O_3
0,5	1,546	2419,3	NEG_QC_3
0,5	1,689	10892,04	NEG_MeOH-1
0,25	1,592	2770,87	NEG_MeOH-1
0,25	1,376	2360,5	NEG_H2O_3
1	1,771	1867,23	POS_H2O_1
0,2	1,705	1767,81	NEG_QC_4
0,2	1,687	1228,52	NEG_H2O_4
0,4	1,782	1901,81	POS_H2O_1
1	1,319	4462,66	POS_MeOH-1
0,09	1,597	1298,34	NEG_MeOH-4
0,17	1,568	6923,46	NEG_MeOH-1
0,25	1,619	11830,48	NEG_EtOH-4

0,79	1,864	68823,27	POS_QC_4
0,67	1,62	1382,48	POS_EtOH-3
0,33	1,763	4125,21	NEG_EtOH-3
1	1,173	1052,81	POS_MeOH-1
0,33	1,367	2518,58	POS_EtOH-1
1	0,876	1980,82	POS_EtOH-1
1	1,424	3640,06	POS_MeOH-3
0,5	1,189	4828,29	NEG_H2O_4

MS1 isotopic spectrum
87.00770:2336 88.01237:0 89.01703:0
148.05991:2584 149.06457:0 150.06924:0
150.05679:3738 151.06146:0 152.06613:0
205.09621:9307 206.10087:0 207.10554:0
166.08521:22554 167.08987:2630 168.09454:0
182.08020:22737 183.08487:3562 184.08954:0
111.01890:22538 112.02357:886 113.02823:0
115.03860:2561 116.04326:0 117.04793:0
104.10620:6434 105.11087:0 106.11554:0
132.10150:1616 133.10617:0 134.11084:0
134.04601:2515 135.05067:0 136.05534:0
134.04630:3619 135.05096:0 136.05563:0
116.06970:3522 117.07437:0 118.07904:0
133.01320:2956 134.01787:0 135.02253:0
298.09521:3014 299.09988:0 300.10455:0
125.03460:12039 126.03927:1007 127.04393:0
89.02340:3391 90.02807:0 91.03273:0
268.10309:26827 269.10776:3511 270.11242:0
303.23169:216928 304.23636:50763 305.24102:6027
303.23160:2672 304.23627:0 305.24093:0
150.04089:10434 151.04556:958 152.05023:0
124.03900:2152 125.04367:0 126.04834:0
377.14520:3843 378.14987:0 379.15454:0
105.01810:2710 106.02276:0 107.02743:0
137.04491:10710 138.04957:0 139.05424:0
267.07239:7845 268.07706:1131 269.08172:0
317.05670:7368 318.06137:2695 319.06604:0
162.11140:4118 163.11607:0 164.12074:0
190.05000:41446 191.05467:2930 192.05934:0
266.08859:7735 267.09326:1274 268.09793:0
883.53583:3529 884.54050:2166 885.54516:777
151.02490:51028 152.02957:2718 153.03424:0
529.15112:2098 530.15579:0 531.16046:0
243.06110:17022 244.06576:1987 245.07043:0
743.48389:4440 744.48855:1701 745.49322:3408
743.48322:2986 744.48788:1313 745.49255:3932
745.50098:8581 746.50564:3473 747.51031:982
745.50061:6519 746.50528:3073 747.50995:814
351.21631:2426 352.22098:729 353.22564:0
118.08580:24155 119.09047:1896 120.09514:0
137.02310:2255 138.02777:0 139.03244:0
122.02360:7316 123.02827:0 124.03293:0
302.30621:2390 303.31088:0 304.31555:0
395.11520:2803 396.11987:1098 397.12454:0
162.07530:23334 163.07997:2111 164.08464:0
146.09100:2497 147.09567:0 148.10034:0
340.28119:2657 341.28586:0 342.29052:0
303.23010:4817 304.23477:1293 305.23944:0
303.23001:2873 304.23468:0 305.23935:0

255.10651:15434 256.11117:2532 257.11584:0
128.03400:12026 129.03866:0 130.04333:0
128.03410:14498 129.03877:0 130.04344:0
527.15802:11967 528.16269:2859 529.16736:0
168.06799:97329 169.07266:5281 170.07733:0
120.07960:1739 121.08427:0 122.08893:0
296.09860:4954 297.10327:669 298.10794:0
103.03910:9013 104.04377:0 105.04844:0
227.20061:56413 228.20528:8060 229.20994:0
277.21609:306114 278.22076:66112 279.22543:7803
365.10440:28886 366.10907:3471 367.11374:1233
337.31021:4210 338.31488:930 339.31955:0
253.21629:90581 254.22096:16801 255.22563:2177
681.41040:1656 682.41507:0 683.41974:0
295.22690:9375 296.23157:1735 297.23623:2167
317.20831:2174 318.21298:0 319.21765:1023
317.20761:7050 318.21228:1250 319.21695:0
331.26309:24857 332.26776:6287 333.27243:0
339.32550:3469 340.33017:0 341.33484:0
367.35641:21427 368.36108:5531 369.36575:873
269.24741:3259 270.25207:0 271.25674:0
309.27802:12439 310.28268:2611 311.28735:0
365.34140:27624 366.34607:7114 367.35074:1667
325.23651:4504 326.24118:0 327.24585:0
696.61420:4208 697.61886:1893 698.62353:0
706.63373:9208 707.63840:4291 708.64306:20229
292.09409:2078 293.09875:0 294.10342:0
449.16510:2494 450.16977:0 451.17444:0
280.12961:7841 281.13428:1803 282.13894:0
315.15479:2624 316.15945:0 317.16412:0
351.24591:3437 352.25058:1186 353.25525:0
273.14511:10103 274.14978:1570 275.15445:0
537.30353:4959 538.30820:1497 539.31286:0
318.29889:2356 319.30356:0 320.30822:0
173.11729:6023 174.12196:0 175.12663:0
174.07561:5972 175.08028:0 176.08494:1806
291.19461:2754 292.19928:0 293.20395:1572
133.04930:4168 134.05397:0 135.05864:0
293.21100:3763 294.21567:727 295.22033:2127
293.21100:24213 294.21567:5432 295.22033:1376
311.22131:3864 312.22598:876 313.23065:0
327.21689:3401 328.22156:0 329.22622:0
298.11270:2766 299.11737:0 300.12204:0
121.02860:2515 122.03327:0 123.03794:0
121.02840:2177 122.03306:0 123.03773:0
291.15610:3370 292.16077:0 293.16543:0
317.10910:8603 318.11377:1852 319.11844:0
309.20621:2323 310.21088:0 311.21554:0
375.21411:12826 376.21878:3096 377.22345:891
317.21069:21426 318.21536:4740 319.22003:2380

282.27802:87250 283.28268:16019 284.28735:1774
320.25449:3140 321.25915:0 322.26382:0
329.23291:2622 330.23758:0 331.24225:0
295.22601:2089 296.23068:0 297.23535:1999
415.20831:5176 416.21298:1954 417.21765:0
193.01511:3818 194.01977:0 195.02444:1415
136.07220:6667 137.07687:0 138.08154:0
292.84409:11393 293.84875:0 294.85342:0

MS/MS spectrum
87.00830:2591
84.04390:3721 102.05560:613 130.04829:1816 144.06490:88 148.05830:139
56.04920:891 61.01090:769 102.05400:208 104.05280:903 114.94690:519 116.98380:94 130.99960:244 133
77.03760:687 86.95130:83 91.05340:1724 107.04790:395 112.95270:82 115.05330:1395 117.05650:951 118
77.03770:6149 79.05390:562 91.05390:1860 93.05700:835 103.05390:6596 120.08040:24861 121.08340:224
65.03770:1439 77.03750:2852 91.05370:9051 95.04860:1581 98.97560:727 107.04950:1149 114.94650:244
41.99740:2475 111.01890:3015
59.01280:131503 60.01630:2856 115.03990:1815 119.03400:2635
45.03220:456 58.06440:1434 60.08090:1417 104.10650:5557
70.06430:342 86.09550:3197 118.97940:181 132.10060:430
59.01270:6165 134.04650:1594
134.04660:1110
70.06450:3599 116.06950:1706
79.95600:11551 133.01241:346
136.06090:2047 298.09839:331
41.99790:1134 60.01610:214 125.03450:1452
89.02320:2903
115.94710:287 119.03420:2187 136.06090:20990 137.06351:1536 152.05650:657 174.96780:530 222.98360
259.24149:2454 303.23160:53650 304.23569:12043
293.17609:1042 303.22900:437
133.01450:430 150.04080:1013
124.03810:2571
186.99370:494 280.93280:214 286.94681:486 288.94449:25 304.89920:148 306.89511:158 368.94919:283 3
79.95610:2183 96.95890:7829 105.01810:416
55.02980:155 82.04080:303 92.02380:462 94.03930:785 98.97430:224 110.03390:1566 119.03530:1909 137
135.02970:2950 267.07120:1929
82.99660:1420 98.97460:51116 102.96750:1338 112.92170:1255 122.97720:3003 123.97680:927 124.97460
82.99630:1083 87.10360:580 98.97510:1731 115.02340:912 116.98540:281 131.00070:598 146.97260:429 1
130.97670:5187 168.06720:992 179.05251:585 187.04311:1421 190.05000:43974 191.05190:2633 192.0468
266.08750:1612
518.19232:655 533.22168:1773 883.53882:3787
108.01910:4665 151.02470:9699
269.99649:371 286.94931:366 347.19550:1824 348.19791:263 370.94800:121 457.28741:612 528.93359:324
243.06030:1691
255.23241:559 279.23071:511 743.48322:3406 744.48352:415
255.23090:340 279.23401:635 743.48523:2626
141.01520:560 242.96609:924 255.23219:5522 256.23489:458 279.23129:3843 745.50012:8327 746.50500:4
255.23230:4896 279.23190:2700 745.49902:6084
351.21580:1753
55.05300:314 58.06460:13643 59.07250:4410 118.08580:26349 119.08840:386
93.03460:1408
59.01280:8503 60.01660:251 78.03360:355 119.03530:1636 122.02300:199
284.29321:985 288.94330:283 293.15009:40 302.30331:1608
273.16821:5550 274.16910:1286 333.18979:2652 362.93381:306 366.92780:61 371.14880:1385 387.11938:1
42.03420:175 60.08010:4421 70.06490:1839 84.04390:1163 98.05990:7088 99.06290:288 116.06980:2756 1
58.06480:1945 59.07280:1526 86.05910:788 86.95350:1645 134.11690:1255 136.02080:714 146.09200:1869
186.99350:491 247.00211:486 266.94730:202 286.95071:34 312.95401:141 329.00400:320 340.28030:3332
91.05280:951 93.06900:180 104.99130:753 143.04581:124 247.00330:246 286.94580:116 293.20691:522 30
287.94830:30 296.29541:1582 303.22971:1548 304.25851:26 306.27451:37

85.02760:339 91.03850:143 93.05410:469 97.02910:168 145.04950:254 163.05820:618 168.06850:1059 190
59.01280:5593 60.01640:192 128.03461:2760
41.99760:1567 59.01280:9178 60.01630:879 61.01680:428 111.01900:2817 128.03349:2493
272.07169:186 365.10199:616 527.15582:9430
168.06841:95997 168.82440:1690 169.07159:5251 170.06480:3234
103.05340:862 120.07840:1095
296.09670:1621
96.95860:2232 103.03830:2165
216.92450:868 223.01871:218 227.20039:14151
277.21600:105875 278.21939:20572
365.10370:11777 366.10440:1424
337.31050:2253
253.21609:38203 254.21950:5391
104.99120:649 186.99541:738 268.99591:1123 433.00119:650 515.00690:1059 589.42749:1859 590.43481:4
216.92410:530 217.00169:616 295.22549:4570 296.23059:80
98.97450:534 256.96371:62 278.95291:342 288.94409:271 290.94281:119 307.22189:127 313.16422:840 31
98.97420:238 290.94199:84 311.21991:324 317.20810:7153 318.20911:1316 319.21939:216
331.26300:4361
244.96470:1976 339.32530:2268
305.02039:433 367.35641:8077 368.35999:2119
269.24759:1668
96.95870:424 126.90410:522 309.27850:4201
365.34091:10007
104.99180:4719 256.96440:543 262.97890:1910 286.94570:2536 288.94170:2188 290.94250:755 311.21790
636.59210:1872
141.01550:159 305.02441:414 387.02609:909 388.35941:1180 390.37189:485 646.61572:5733 647.61749:22
286.94730:582 289.94510:262 292.09698:1155
284.97021:165 305.23050:335 327.21490:2655 328.21719:955 329.21161:129 365.25320:233 387.23291:92
89.04100:414 98.97460:200 132.95750:495 184.95790:188 186.95770:375 187.99580:201 246.97951:230 25
278.95190:189 288.94461:322 311.21509:394 313.16641:781 315.15640:1705
98.97480:108 186.99490:148 268.99780:346 278.95160:126 298.90009:86 351.00250:51 351.21390:749 351
114.94720:73 132.95779:105 142.94701:201 174.96790:344 182.96150:319 184.96060:175 242.98190:198 2
104.99130:472 268.99680:1055 270.00269:510 286.94629:112 288.94241:409 370.94861:222 481.22891:104
104.99140:405 219.01630:182 256.26379:170 309.20541:383 311.21841:79 313.16000:837 318.29901:3733
173.11780:1888
174.07540:2135
141.01559:765 216.92310:342 218.92101:528 223.01820:261 227.20070:6305 291.19690:305 293.17670:139
59.01250:4391 133.04940:1641
216.92360:459 218.92020:370 293.21011:2124 295.22659:1239
218.92050:815 275.20111:592 293.21121:7616 294.21429:1104
240.96880:329 242.96449:250 311.22229:1131
126.90350:1227 327.21710:461
98.97440:252 135.02930:536 152.05521:3533
59.01280:124582 60.01600:4041
59.01280:116626 59.05680:1188 60.01630:3071 119.03430:2365
143.04539:185 198.97549:168 291.15451:3631 292.16061:384
77.03810:327 79.05370:675 81.06980:165 91.05310:2344 93.06910:968 105.06890:1215 121.10060:392 133
119.04950:1760 126.90400:453 183.01140:10248 232.97411:500 240.96860:846 244.96530:202 311.16751:1
141.01559:299 216.92290:265 375.21390:2148
126.90300:198 232.97250:390 240.96870:432 244.96291:52 312.81979:88 317.21100:4284

69.06890:711 79.05300:1007 81.06900:1389 83.08470:2956 91.05320:768 93.06840:1824 95.08530:3232 97
98.97370:400 256.96069:345 262.97900:301 278.95169:334 286.94711:469 288.94290:126 311.19910:684 3
329.23230:1965
143.04620:640 180.97710:143 263.10501:131 277.21759:690 279.22879:567 286.94739:253 288.94370:224
219.09911:2315 415.20959:888
54.98490:909 55.93410:373 70.95700:1302 72.93610:3793 82.99680:5861 83.99840:564 84.99550:998 98.97
86.95200:1421 88.96860:1343 118.97750:1119 136.07110:2050
92.92670:1911 94.92380:1814 210.83940:393 288.77509:323 290.84619:683 292.84509:539

POS_EtOH-1	POS_EtOH-2	POS_EtOH-3	POS_EtOH-4	POS_H2O_1	POS_H2O_2	POS_H2O_3
0	1046	888	1202	10059	16477	9148
5579	6360	5333	5281	3544	10745	13462
0	0	0	0	38278	16039	20588
0	0	0	917	29402	25968	25946
3831	4873	5996	5831	68670	83983	91840
7189	6704	7362	6575	63591	62378	71221
37176	42988	37217	40657	139145	144447	155318
0	0	0	0	5212	1469	3182
24181	27797	21936	24302	48966	50275	42864
7915	8806	7596	6876	33384	59793	62339
10447	9494	9238	8968	0	0	0
16436	15520	15410	16697	0	0	0
9269	11733	10005	13625	1685	7121	14352
0	0	0	0	6595	15457	13256
7885	6118	7651	5972	0	0	0
7704	9232	7229	8395	41511	34016	45260
12903	7824	8314	9095	68515	30376	56643
159637	178821	163708	184876	0	21812	0
568025	545024	570565	544411	8175	8695	7926
14286	12534	13441	13990	0	0	0
3096	6129	3529	3923	24817	28622	27701
7504	12660	7487	12390	13479	22937	21062
5321	3367	3909	3492	10469	8084	10775
4792	4385	3935	4353	4167	7349	5723
25704	16725	26041	24957	127065	121113	133340
52754	36998	39990	43440	0	116608	0
17773	23090	18961	19392	8166	11837	11923
8418	10236	8482	10187	7792	11114	10875
182832	183957	182628	189328	211836	205348	210325
27222	22388	24429	24528	0	28160	0
4482	2392	0	0	113783	0	15968
35786	35282	33232	36073	140735	145352	154052
4530	3842	3097	4345	0	0	0
18350	17343	17206	18578	0	96813	0
0	28255	0	27585	0	0	0
20081	282	24072	27585	0	0	0
0	60283	0	56118	0	0	0
49997	1092	53696	56118	0	0	0
15038	23695	24542	25402	23874	39250	37877
60238	72021	58343	69000	66040	73906	78259
3838	7883	4787	6082	7101	16171	9527
9918	10597	10202	9912	15188	16214	15607
4937	5344	5399	5693	0	0	0
13645	12822	11626	12861	0	0	0
27876	31878	28367	28923	50682	54863	54632
3879	1966	2826	4693	3409	4705	4055
4382	1744	0	0	0	520	631
19048	19248	21545	20926	0	0	0
4699	3734	1938	4894	0	0	0

17891	22919	19542	20429	27772	31287	32256
4662	4413	5845	6557	25508	37344	37430
34122	35176	33674	36257	0	0	0
0	0	0	0	14959	33778	12552
311134	335946	300545	331492	394298	401697	396301
0	0	0	0	23537	22704	25046
3639	3029	2761	3027	13087	13247	13383
25677	26264	24937	24689	22426	30463	32161
176550	144043	163798	160720	19715	16775	14801
897857	863927	908918	885908	20976	20259	19389
5275	6529	5139	5517	106029	39093	23869
15701	15581	17163	15603	0	0	0
295377	291272	296560	287647	8778	8755	7588
5181	0	2905	3917	0	0	0
70096	72715	71446	75395	8796	8388	8987
8720	23196	9396	10154	5332	7250	4242
32181	32717	32140	30066	9819	13098	12355
54998	52927	54846	49826	0	0	0
7899	7656	6955	7488	839	0	0
127185	267358	160096	312818	9609	29118	18924
16028	7099	12138	8523	0	357	397
32894	28670	35880	33159	1919	1321	1289
105326	95756	103079	100708	811	0	0
12912	12457	12807	11993	0	0	0
13427	14079	14346	14856	0	0	0
40767	12675	29655	17343	0	0	0
0	0	0	0	4919	6168	6660
6955	5115	6634	4305	0	0	0
18125	0	0	0	33363	74975	76726
7303	6578	9473	4782	0	0	0
5434	0	0	0	11648	75924	47078
39513	41194	36759	45366	33973	43143	36061
13807	12585	12841	13962	0	0	0
5262	4379	0	0	1058	0	1029
0	759	0	0	19150	15614	22144
12715	11993	9552	10744	12970	14167	15195
13008	12185	12914	13087	16964	17771	15482
8377	9090	7554	8974	8442	9893	11114
31701	29087	25996	30757	9193	14922	8357
111071	109665	110644	110431	44336	57023	48498
18377	20472	21608	18496	17558	24312	21101
11624	12352	10738	11876	22312	21634	21031
937	0	0	0	7906	12190	8555
10308	7048	7727	7810	0	0	1219
3284	1032	819	1566	4786	3570	6906
12079	12102	12941	11469	17147	16070	17919
29133	30558	28811	28714	2885	6415	2606
8440	16672	9319	13251	0	0	0
66269	65879	67018	70334	26604	36412	26525
91061	90254	86922	90648	16021	25247	16353

645048	383164	613878	171214	7001	3378	0
4789	825	5512	0	0	0	0
24119	22796	21206	24003	50616	42218	41765
9365	3043	4530	4224	0	0	0
14351	11998	13226	12960	0	0	0
14140	17513	12210	14907	0	0	0
19508	21176	19116	17174	21497	20042	20336
19774	19397	19545	17971	17869	16963	16945

POS_H2O_4	POS_MeOH-1	POS_MeOH-2	POS_MeOH-3	POS_MeOH-4
13476	1400	0	0	849
2216	4053	3851	5842	4185
11514	0	0	0	0
23404	0	1851	914	0
71224	6851	4911	6950	5679
70140	9852	9675	9155	9260
157494	66706	65004	68170	72769
3286	0	0	0	0
50553	22171	20115	26543	25949
52413	7975	11356	9669	10400
0	11709	11649	11716	11573
0	16482	15353	16616	17156
8204	8839	8724	9400	8602
10851	0	0	0	0
0	5713	6634	8569	7058
42028	9143	9188	9369	8588
42337	10574	8062	16869	9622
0	179428	181519	190493	189804
7706	592640	585322	623673	613855
0	13906	14334	14465	14484
30285	10339	11022	10800	12312
21729	13292	10533	14421	15962
6449	3061	5985	4997	4103
4336	3821	3148	4531	4771
136451	30257	29777	32296	31854
0	57579	55252	52292	54339
13827	22805	20955	26234	28633
9792	9960	7056	8713	10091
222339	153167	143344	159724	167715
0	26108	26005	25221	24434
17088	30118	31819	0	0
156036	38228	37543	37305	39044
0	3787	4811	3240	4143
0	60745	59996	59667	60084
0	18977	16664	18809	20840
0	18977	16664	0	0
0	35411	33836	36459	40775
0	35411	33836	0	0
20052	25759	15818	23774	25126
77963	66073	63783	76895	76681
16425	6325	2745	5038	5236
14243	10197	10777	10985	11390
0	5113	4940	2878	2023
0	13619	12115	10606	9459
59433	25972	21926	23801	23574
5121	2078	2298	2629	1074
1107	2379	3090	10217	733
0	21269	20596	19146	19398
0	5509	2968	3470	4722

32381	21411	17482	23875	24771
30017	63356	64336	64836	70698
0	0	0	0	0
16732	0	0	1510	383
409613	239251	230093	281084	281956
20327	0	0	0	0
11796	3660	3038	3332	3320
29963	27767	27282	25546	26278
13608	161585	163698	160002	153638
18360	911206	939479	952878	931664
64121	5951	7201	6630	5214
0	10721	11074	10131	11922
7821	301921	322590	336772	323601
0	3999	4347	4286	2803
7757	78636	79083	73347	76350
4931	12113	10345	9633	12455
13031	30998	33192	33637	33678
0	52495	53502	68011	62774
0	3977	2169	2673	2777
11875	132959	36869	16849	23781
329	6466	9572	7441	7079
871	29751	31903	32759	30973
0	50447	33328	21468	31025
0	12439	14098	8599	9388
0	13170	11988	12840	13556
0	3494	5734	2433	2175
2247	0	0	3148	0
0	4674	5944	6397	5280
68296	0	0	40326	0
0	5689	6133	7453	6974
23340	0	0	7921	0
41617	46736	44150	41825	45252
0	15097	14272	14664	15505
896	4670	3917	13630	1880
20293	0	702	693	706
15108	9333	9523	11034	10889
19844	13463	13816	13357	12465
11082	8507	7469	9178	9032
9323	31432	32638	29596	21429
46340	115900	118747	112126	113323
17150	20502	21176	18467	20972
22228	10957	10062	9995	11653
8874	1975	0	1236	1361
0	7463	8706	7908	8157
5237	1837	1473	3718	2865
16381	12676	14219	13980	13017
3558	30142	32891	27963	27594
0	17610	11920	10736	12198
27553	72196	72083	66674	68611
18889	98520	94578	97243	98744

3526	292509	606829	783967	409193
0	0	3389	6286	0
42136	22665	22655	23274	23367
1131	5307	3770	2462	2463
0	11970	14632	11567	13109
0	0	0	0	0
20593	22435	21189	20556	20587
17939	19396	19258	20308	20282
